# Dynamic activation of lytic cell death-related programs identifies CD14 as a candidate hub gene associated with secondary injury after spinal cord injury

**DOI:** 10.3389/fimmu.2026.1841784

**Published:** 2026-07-06

**Authors:** Shenglong Wang, Ruoxiang Mei, Wenting Xu, Xiaochen Su, Menghao Teng, Xiaoxiao Lou, Chen Zhang, Shenghua Guo, Yingang Zhang

**Affiliations:** 1 Department of Orthopedics, The First Affiliated Hospital of Xi’an Jiaotong University, Xi’an, China; 2 The Second Affiliated Hospital of Xi’an Jiaotong University, Xi’an, China

**Keywords:** CD14, ferroptosis, lytic cell death, necroptosis, pyroptosis, spinal cord injury

## Abstract

**Background:**

Secondary spinal cord injury (SCI) involves persistent inflammation, oxidative stress, and multiple forms of programmed cell death. However, the dynamic activation of lytic cell death-related programs and their key regulatory nodes during SCI progression remain unclear.

**Methods:**

Public transcriptomic datasets were analyzed using single-sample gene set enrichment analysis (ssGSEA) to assess pyroptosis-, necroptosis-, and ferroptosis-related transcriptional activities, and integrated lytic cell death-related indices were constructed. Differential expression analysis, weighted gene co-expression network analysis, functional enrichment, and multiple machine learning models were combined to identify candidate hub genes associated with lytic cell death-related signatures. Key findings were further evaluated using an external human SCI-related cohort, RT-qPCR and immunofluorescence validation in a rat SCI model, and published single-cell RNA-seq data.

**Results:**

Pyroptosis-, necroptosis-, and ferroptosis-related transcriptional activities were all increased after SCI, with activation beginning in the acute phase and persisting over time. Enrichment analyses showed that lytic cell death-associated genes were mainly involved in inflammatory responses, immune regulation, myeloid activation, and related signaling pathways. Integrated multi-model analysis identified CD14 as the most robust candidate hub gene associated with the lytic cell death index. External validation in a human SCI-related peripheral blood cohort, RT-qPCR and immunofluorescence validation in a rat SCI model, and single-cell reanalysis further supported the upregulation of CD14 and its association with myeloid inflammatory activation and pyroptosis-, necroptosis-, and ferroptosis-related signatures.

**Conclusions:**

Lytic cell death-related programs are dynamically and persistently activated after SCI and are closely associated with immune-inflammatory responses during secondary injury. CD14 was identified as a candidate hub gene associated with myeloid inflammatory activation and lytic cell death-related signatures. However, the current findings are primarily associative, and further functional studies are required to determine whether CD14 directly modulates lytic cell death-related pathways and contributes to secondary injury progression after SCI.

## Graphical Abstract

**Figure:**
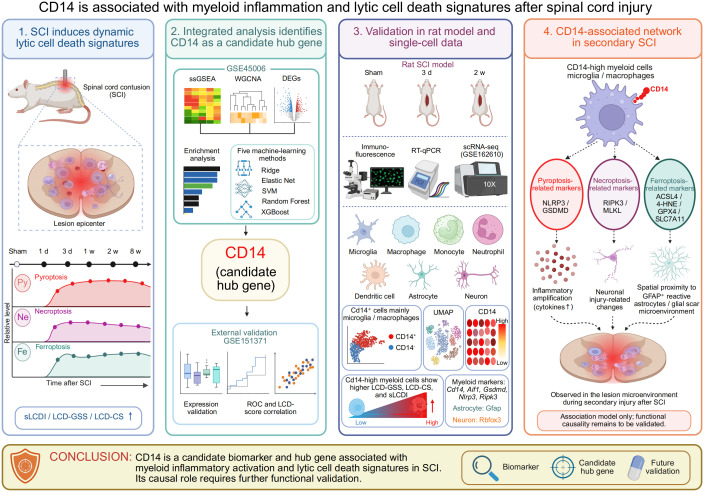
CD14-associated myeloid inflammation and lytic cell death signatures after spinal cord injury. Created in BioRender. Wang, S. (2026). https://BioRender.com/55m178q.

## 1 Introduction

Spinal cord injury (SCI) is a devastating form of central nervous system trauma that can result in long-term or even lifelong motor, sensory, and autonomic dysfunction. It is characterized by a high rate of disability, a substantial socioeconomic burden, and limited therapeutic options (1–7). Although notable advances have been made in acute care, perioperative management, and rehabilitation strategies in recent years, overall functional recovery after SCI remains limited, and clinical outcomes are still largely determined by injury severity and subsequent secondary pathological changes (2–4). Accordingly, the central scientific challenge in SCI research is no longer confined to the primary mechanical insult itself, but has shifted toward understanding and intervening in the persistent and severe secondary injury processes that follow.

In contrast to the immediate tissue destruction caused by the primary mechanical impact, secondary injury represents a dynamic and progressive cascade that extends from the acute phase through the subacute stage and even into the chronic phase. This process involves multiple interconnected pathological events, including innate immune activation, redox imbalance, blood–spinal cord barrier disruption, glial reactivity, extracellular matrix remodeling, and the sustained loss of neurons and glial cells (5–11). These pathological responses do not occur in isolation; rather, they collectively drive injury progression through inflammatory amplification, disrupted intercellular communication, persistent deterioration of the local microenvironment, and stabilization of scar architecture, ultimately limiting axonal regeneration and neural circuit reconstruction (5, 6, 8–11). Despite the continuous development of therapeutic strategies targeting immune modulation, blood–spinal cord barrier protection, cell-based therapies, and drug repurposing, there is still a lack of an integrative explanatory framework that can account for both the temporal dynamics and network-level complexity underlying the persistent amplification, stage-specific progression, and eventual chronic consolidation of secondary SCI pathology (6–11).

In recent years, lytic cell death has increasingly been recognized as a critical pathological hub linking tissue destruction to inflammatory amplification. This concept emphasizes that, during the process of cell death, disruption of membrane integrity, leakage of intracellular contents, and release of damage-associated molecular patterns collectively amplify local inflammatory responses and tissue injury (12–18). In the context of SCI, pyroptosis, necroptosis, and ferroptosis are currently the three most extensively studied lytic cell death-related programs. Although these processes differ in their molecular execution mechanisms, all of them can promote sustained deterioration of the post-injury microenvironment by inducing membrane damage, enhancing inflammatory mediator release, triggering lipid peroxidation, and aggravating oxidative stress (15–23). More importantly, accumulating evidence suggests that these three forms of cell death do not occur in isolation. Instead, inflammasome signaling, the RIPK1/RIPK3/MLKL cascade, ROS accumulation, mitochondrial dysfunction, and lipid peroxidation can cross-activate one another, thereby forming an interconnected inflammatory cell death network (17–23). Taken together, these findings indicate that cell death after SCI should no longer be understood simply as several parallel single-pathway events, but rather as an integrated pathological axis that spans multiple stages and actively participates in remodeling the injured microenvironment.

However, current research on lytic cell death in SCI still has two major limitations. First, previous studies have largely focused on a single mode of cell death or a few canonical molecules, primarily addressing whether a specific pathway is involved in SCI. In contrast, the relative contributions, overall activity changes, and synergistic interactions of pyroptosis, necroptosis, and ferroptosis across different temporal stages remain insufficiently quantified and dynamically compared in a systematic manner (18–23). Second, evidence remains limited regarding which molecules are capable of simultaneously linking inflammatory activation with multiple lytic cell death programs and functioning as shared interfaces or network hubs during secondary injury (24–30). In particular, for candidate molecules associated with injury sensing, innate immune responses, and myeloid cell biology, there is still a lack of systematic studies integrating transcriptomic dynamic analysis, co-expression network modeling, machine learning-based screening, and histological validation. This gap limits our ability to identify the key drivers of secondary SCI injury and also constrains the transition of intervention strategies from single-pathway inhibition to network-level regulation.

Based on this background, the present study was designed from a systems biology perspective to address whether lytic cell death constitutes an important integrated pathological axis in secondary SCI injury. First, pyroptosis-, necroptosis-, and ferroptosis-related gene sets were integrated from transcriptomic data, and single-sample gene set enrichment analysis (ssGSEA) was used to construct temporal activity profiles of lytic cell death at different time points after SCI. A composite lytic cell death index was then generated to characterize the dynamic changes of this pathological program following SCI. Subsequently, differential expression analysis, weighted gene co-expression network analysis, and multiple machine learning models were combined to identify candidate hub genes most closely associated with lytic cell death, and these findings were further validated in an independent external cohort and in a rat SCI model. Given the established biological role of CD14 in innate immune recognition, myeloid cell activation, and central nervous system injury responses, we further hypothesized that CD14 may represent a candidate molecular hub associated with myeloid inflammatory activation and lytic cell death-related transcriptional programs after SCI.

## 2 Materials and methods

### 2.1 Calculation of lytic cell death-related pathway activity scores

The preprocessed gene expression matrix and sample grouping information for the GSE45006 dataset were imported, and samples were ordered according to the Sham group and the post-injury time points of 1 day, 3 days, 1 week, 2 weeks, and 8 weeks after spinal cord injury. After confirming the consistency between sample annotations and the expression matrix, gene annotation information was standardized. Based on the Gene Ontology Biological Process and Reactome pathway annotations for Rattus norvegicus in the MSigDB database, gene sets related to pyroptosis, necroptosis, and ferroptosis were constructed. The pyroptosis gene set was further supplemented with several canonical core molecules, including Casp1, Gsdmd, Nlrp3, Pycard, Il1b, and Il18. Single-sample gene set enrichment analysis (ssGSEA) was then performed using the GSVA package to calculate enrichment scores for pyroptosis-, necroptosis-, and ferroptosis-related pathways in each sample (31).

To more comprehensively characterize lytic cell death-related transcriptional activity, three complementary composite indicators were retained: the Standardized LCD Index (sLCDI), the LCD Gene Set Score (LCD-GSS), and the LCD Composite Score (LCD-CS). LCD-GSS was defined as the ssGSEA score calculated from a single merged gene set containing pyroptosis-, necroptosis-, and ferroptosis-related genes, and was used to reflect the global enrichment pattern of the overall lytic cell death-related transcriptomic program. LCD-CS was defined as the arithmetic mean of the three individual ssGSEA scores for pyroptosis, necroptosis, and ferroptosis, and was used to represent the average activity level of the three major lytic cell death-related programs. sLCDI was defined as the mean of the Z-score-standardized ssGSEA scores for pyroptosis, necroptosis, and ferroptosis, and was used as a standardized integrative index to capture the coordinated variation of these three programs while reducing the influence of differences in score scale.

These three indicators reflect distinct but complementary aspects of lytic cell death-related transcriptional activity. LCD-GSS emphasizes the global enrichment of a merged lytic cell death-related gene set, LCD-CS reflects the average activity level across the three major lytic cell death programs, and sLCDI highlights their relative coordinated activation after standardization. Importantly, the individual pathway scores and the composite indices were used for different purposes. The individual pyroptosis, necroptosis, and ferroptosis scores were retained to describe pathway-specific activation patterns and temporal differences after SCI, whereas the composite indices were designed to summarize the integrated lytic cell death-related transcriptional state. Therefore, the composite indices were not intended to replace the individual pathway scores, but to provide complementary information for downstream network-level analyses, including WGCNA, candidate gene screening, and hub-gene prioritization.

Finally, violin plots, principal component analysis (PCA), and heatmaps were used to visualize the dynamic changes in these pathway activity scores across different post-injury time points.

### 2.2 Identification of key sLCDI-related modules by weighted gene co-expression network analysis

Weighted gene co-expression network analysis (WGCNA) was performed using the normalized expression matrix of GSE45006. After transposing the expression matrix into a sample-by-gene format, the top 5,000 genes with the highest expression variance were retained to improve network stability. The goodSamplesGenes function was then applied to identify and remove outlier genes and samples. The previously calculated Standardized LCD Index (sLCDI) values were incorporated as trait data, with sample order matched exactly between the expression matrix and trait matrix.

The pickSoftThreshold function was used to assess scale-free topology fit and mean connectivity across a series of soft-thresholding powers, and an appropriate soft-thresholding power was selected for construction of the adjacency matrix and topological overlap matrix. Gene co-expression modules were then identified using the dynamic tree-cutting algorithm. In WGCNA, module assignment was based on the similarity of gene co-expression patterns and topological overlap, rather than on the direction of association between individual genes and external traits. Gene–gene correlations were transformed into an adjacency matrix using a soft-thresholding power and then into a topological overlap matrix, followed by hierarchical clustering and dynamic tree cutting for module detection (32–34). After modules were defined, module eigengenes were calculated and correlated with sLCDI and other lytic cell death-related scores. Therefore, modules were prioritized according to the absolute strength of their module–trait correlations. For modules negatively correlated with sLCDI, genes with negative module membership but positive gene significance were interpreted as sLCDI-activated anti-eigengene genes, whereas genes with positive module membership but negative gene significance were interpreted as sLCDI-suppressed eigengene-aligned genes.

### 2.3 Construction of the candidate gene set and functional enrichment analysis

Using the GSE45006 dataset, genes from the WGCNA module most strongly associated with the Standardized LCD Index (sLCDI) were first extracted. Based on the normalized expression matrix, differential expression analysis between the injury groups and the Sham group was then performed using the limma package, with adjusted P value (adj.P.Val) < 0.05 and |log_2_FC| ≥ 1 as the thresholds for identifying differentially expressed genes (35). The intersection between the genes in the module most strongly associated with sLCDI and the differentially expressed genes was defined as the sLCDI-related candidate gene set. These candidate genes were subsequently converted into Entrez IDs, and clusterProfiler together with ReactomePA was used to perform Gene Ontology (GO), Kyoto Encyclopedia of Genes and Genomes (KEGG), and Reactome enrichment analyses. In addition, Hallmark, ImmuneSig, and DrugPerturb enrichment analyses were conducted based on the MSigDB database. To further characterize the biological context associated with lytic cell death activity, a preranked gene list was generated according to the Spearman correlation coefficients between all genes and sLCDI, followed by Hallmark-based gene set enrichment analysis. This approach was used to systematically evaluate the potential involvement of sLCDI-related candidate genes in inflammatory responses, immune regulation, and lytic cell death-associated biological processes.

### 2.4 Identification of sLCDI-related hub genes using multiple machine learning models

The previously defined sLCDI-related candidate gene set was used as the input feature set for machine learning analysis. Using the GSE45006 dataset, a binary classification training set was constructed in which the Sham group was defined as the control group and samples from all post-injury time points were combined into a single Injury group. After centering and scaling of the candidate gene expression matrix, five machine learning algorithms, including Ridge regression, Elastic Net, support vector machine (SVM), random forest (ranger), and XGBoost, were applied for feature selection, and feature importance was evaluated using repeated cross-validation. The gene importance rankings generated by the different models were subsequently integrated, and the average rank (AvgRank) was calculated to establish a multi-model consensus ranking. This strategy was used to reduce model-specific bias and to identify the most robust and representative hub genes associated with sLCDI.

### 2.5 Visualization analysis of the expression patterns of the top 10 candidate genes

The expression patterns of the top 10 candidate genes identified by the integrated multi-machine learning analysis were further evaluated in the GSE45006 dataset. First, the expression matrix of these 10 genes was extracted across all samples, which were then classified into the Sham and Injury groups, with the Injury group including samples collected at D1, D3, W1, W2, and W8. After gene-wise normalization of the expression matrix, a heatmap was generated to visualize the overall expression patterns of the candidate genes across samples.

In addition, violin plots combined with box plots were generated for each gene to compare expression differences between the Sham and Injury groups. Statistical significance was assessed using the Wilcoxon rank-sum test, and P values from the 10 candidate-gene comparisons were adjusted using the Benjamini–Hochberg false discovery rate (FDR) method. This analysis was performed to validate the expression consistency of the candidate genes identified by the multi-model machine learning framework at both the global and single-gene levels, and to provide visual support for subsequent hub gene determination.

### 2.6 Expression characteristics of the hub gene and its association with lytic cell death-related scores

The expression level of the hub gene was further extracted and compared across different post-injury time points and between different lytic cell death (LCD) subgroups, with the high-LCD-CS and low-LCD-CS groups defined according to the median value of the LCD Composite Score (LCD-CS). Spearman correlation analysis was then performed to evaluate the associations between Cd14 expression and the scores of pyroptosis, necroptosis, ferroptosis, LCD Gene Set Score (LCD-GSS), and LCD Composite Score (LCD-CS). Bar plots and scatterplots with fitted regression lines were used to visualize the strength and direction of these associations. This analysis was designed to clarify the relationship between *Cd14* and lytic cell death from the perspectives of both temporal expression dynamics and pathway-level correlations.

### 2.7 External validation of hub gene expression and its association with lytic cell death-related scores in GSE151371

To evaluate the clinical relevance of the findings from the discovery cohort in an independent human dataset, GSE151371 was obtained from the Gene Expression Omnibus database. GSE151371 is a human peripheral white blood cell RNA-seq dataset generated on the Illumina HiSeq 4000 platform. The dataset contains 58 samples, including 10 healthy controls, 10 non-CNS trauma controls, and 38 patients with traumatic SCI. Because this dataset was derived from human clinical samples rather than an experimental animal model, no experimental SCI modeling method was involved. Peripheral blood samples were collected from patients with acute traumatic SCI as early as possible after hospital admission. In the present study, healthy control and SCI samples were retained for external validation. The expression matrix of CD14 was extracted, and differential expression between the HC and SCI groups was assessed. Receiver operating characteristic (ROC) curve analysis was further performed to evaluate the discriminatory ability of CD14 for distinguishing SCI from HC samples.

Using the same analytical strategy as in the discovery cohort, single-sample gene set enrichment analysis (ssGSEA) was performed for pyroptosis-, necroptosis-, and ferroptosis-related gene sets to obtain pathway-associated transcriptional scores for each sample. Composite lytic cell death indicators were then calculated, including the LCD Gene Set Score (LCD-GSS), defined as the ssGSEA score of the merged lytic cell death-related gene set, and the LCD Composite Score (LCD-CS), defined as the mean of the pyroptosis, necroptosis, and ferroptosis scores. Differences in these scores between the HC and SCI groups were assessed using the Wilcoxon rank-sum test, and Spearman correlation analysis was used to evaluate the associations between CD14 expression and each lytic cell death-related indicator. Because GSE151371 lacks matched fine-grained post-injury time-point information comparable to GSE45006, it was used to provide external human validation of CD14 expression and its association with lytic cell death-related transcriptional signatures, rather than to validate the temporal dynamics observed in the discovery cohort. This human dataset was selected because SCI is ultimately a human disease, and validation in human clinical samples provides important translational support. However, because injured human spinal cord tissue is extremely difficult to obtain, this dataset was derived from peripheral white blood cells rather than spinal cord tissue, which should be considered when interpreting the validation results.

### 2.8 Rat spinal cord injury model and immunofluorescence assessment of CD14-associated spatial patterns

To examine the histological association between CD14 and lytic cell death-related markers identified by the bioinformatics analyses, a rat spinal cord contusion model was established. Spinal cord tissue samples were collected from the Sham group and at 3 days and 2 weeks after injury. The 3-day time point was selected to represent the acute phase, during which lytic cell death-related transcriptional activity was markedly activated, whereas the 2-week time point was selected to represent the subacute phase characterized by sustained inflammatory and glial responses. These time points were used for tissue-level validation of the acute-to-subacute associations identified in the transcriptomic analysis. Double immunofluorescence staining was performed to assess the spatial relationships of CD14 with Iba1, GSDMD, p-MLKL, and GFAP. In addition, NeuN and 4-HNE staining was performed to evaluate neuronal lipid peroxidation-related injury. Representative whole-lesion images and corresponding higher-magnification regions were acquired to show both the overall lesion distribution and local cellular-level staining patterns. Relative fluorescence intensity profiles and spatial distribution analyses were further performed to evaluate the spatial association between CD14 and inflammatory or lytic cell death-related markers during SCI progression. These analyses were used to provide histological support for the association between CD14 expression, myeloid inflammatory activation, and lytic cell death-related molecular signatures, rather than to establish a causal regulatory role for CD14.

### 2.9 RT-qPCR assessment of *Cd14* and lytic cell death-related gene expression

To further examine the expression patterns of *Cd14* and lytic cell death-related genes after spinal cord injury (SCI), a rat spinal cord contusion model was established. Spinal cord tissues from the lesion center and adjacent segments were collected from the Sham group and at 3 days and 2 weeks after injury. These two post-injury time points were chosen to examine representative acute and subacute transcriptional changes in Cd14 and lytic cell death-related genes, rather than to validate the full long-term temporal trajectory observed in the discovery transcriptomic dataset. Total RNA was extracted using TRIzol reagent and reverse-transcribed into cDNA. RT-qPCR was then performed to measure the expression levels of *Cd14*, pyroptosis-related genes (*Gsdmd* and *Nlrp3*), necroptosis-related genes (*Ripk3* and *Mlkl*), ferroptosis-related genes (*Acsl4*, *Gpx4*, and *Slc7a11*), as well as *Aif1* and *Gfap*. The primer sequences are provided in **Supplementary Table 1**. Gapdh was used as the internal control. Relative gene expression was calculated using the 2^-ΔΔCt^ method and visualized by bar plots, row-scaled Z-score heatmaps, and temporal trend plots. This analysis was performed to assess whether *Cd14* and lytic cell death-related genes exhibited coordinated expression changes after SCI.

### 2.10 Single-cell data reanalysis and marker validation

Publicly available single-cell RNA-seq data from GSE162610 were reanalyzed using Seurat. The official cell-type annotations and injury time-point information were retained, including uninjured spinal cord and samples collected at 1, 3, and 7 days post-injury (dpi). *Cd14*-positive cells were defined as cells with *Cd14* expression > 0, and their proportions were calculated across different cell types and injury time points. Myeloid cells were defined to include microglia, macrophages, monocytes, neutrophils, dendritic cells, and dividing myeloid cells. Lytic cell death (LCD)-related scores, including the Standardized LCD Index (sLCDI), LCD Gene Set Score (LCD-GSS), and LCD Composite Score (LCD-CS), were calculated and compared between *Cd14*-low and *Cd14*-high myeloid cells, as well as across different injury time points. In addition, the cellular distribution and expression patterns of RT-qPCR/immunofluorescence-related marker genes, including *Cd14*, *Aif1*, *Gsdmd*, *Nlrp3*, *Mlkl*, *Ripk3*, *Acsl4*, *Gpx4*, *Slc7a11*, *Gfap*, and *Rbfox3*, were examined across annotated cell types.

### 2.11 Statistical analysis

Statistical analyses were performed using R and GraphPad Prism software (version 9.02; GraphPad Software Inc., USA), as appropriate. For transcriptomic analyses, comparisons between two groups were performed using the Wilcoxon rank-sum test, and P values were adjusted using the Benjamini–Hochberg false discovery rate (FDR) method where applicable. Spearman correlation analysis was used to assess associations between CD14/*Cd14* expression and lytic cell death-related transcriptional scores. Receiver operating characteristic (ROC) curve analysis was performed to evaluate the discriminatory ability of CD14 in the external validation cohort. For RT-qPCR data, results are presented as mean ± standard deviation (SD), and comparisons among the Sham, 3-day, and 2-week groups were performed using one-way analysis of variance (ANOVA). For single-cell analyses, comparisons between Cd14-low and Cd14-high myeloid cells were performed using the Wilcoxon rank-sum test, with Benjamini–Hochberg FDR correction where applicable. A P value or adjusted P value < 0.05 was considered statistically significant.

## 3 Results

### 3.1 Lytic cell death-related programs exhibit time-dependent dynamic activation after spinal cord injury

**Figure 1:**
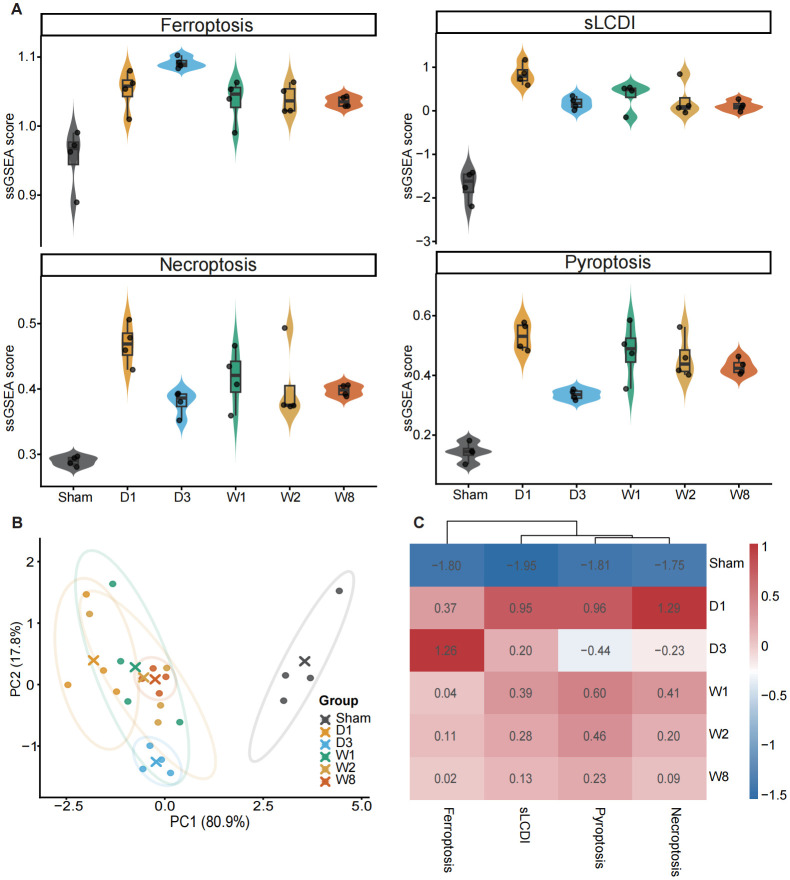
Temporal dynamics of lytic cell death-related signatures after spinal cord injury. GSE45006 included 4 Sham samples and 20 SCI samples, with 4 biological replicates at each post-injury time point. ssGSEA scores were calculated for each sample. Individual pathway scores were used to show pathway-specific temporal patterns, whereas sLCDI was used to summarize the standardized coordinated activation of the three lytic cell death-related programs. **(A)** Violin plots showing the ssGSEA scores of ferroptosis, pyroptosis, necroptosis, and the Standardized LCD Index (sLCDI) in the Sham group and at different time points after spinal cord injury (D1, D3, W1, W2, and W8). **(B)** Principal component analysis (PCA) based on the four pathway activity scores, showing the separation between the Sham group and the post-injury groups. **(C)** Heatmap showing the group-wise mean ssGSEA scores of the four signatures across different time points.

ssGSEA revealed that, compared with the Sham group, lytic cell death-related programs were activated at all post-injury time points after spinal cord injury, although their temporal patterns were not identical (**Figure 1A**). Specifically, pyroptosis, necroptosis, and sLCDI were markedly increased as early as 1 day after injury, indicating that the acute phase represents the most active stage of the lytic cell death response. In contrast, ferroptosis reached its peak at 3 days after injury, suggesting a slightly delayed activation relative to the other forms. Thereafter, although the activity of these pathways declined from 1 to 8 weeks compared with the acute phase, their levels remained overall higher than those in the Sham group, indicating that lytic cell death not only participates in the early secondary injury after SCI but may also continue to influence subsequent pathological progression.

Principal component analysis (PCA) showed a clear separation between the Sham group and the samples from all post-injury time points along PC1, with PC1 and PC2 explaining 80.9% and 17.8% of the total variance, respectively (**Figure 1B**), suggesting substantial remodeling of lytic cell death-related molecular features after SCI. The heatmap further showed that the D1 group exhibited the highest activity of sLCDI, pyroptosis, and necroptosis, whereas ferroptosis was most prominent in the D3 group. Although the W1, W2, and W8 groups showed a gradual decline, their overall levels remained higher than those of the Sham group (**Figure 1C**), further supporting the persistent and stage-dependent activation of these programs.

After combining all post-injury samples into a single Injury group, the heatmap showed that FERROPTOSIS, PYROPTOSIS, NECROPTOSIS, and LCD-GSS were globally higher in the Injury group than in the Sham group (**Supplementary Figure 1A**). Consistently, violin plots confirmed that the Injury group had significantly increased scores for FERROPTOSIS, NECROPTOSIS, PYROPTOSIS, LCD-GSS, and LCD-CS (**Supplementary Figures 1B–F**), further validating the broad activation of lytic cell death-related programs after SCI. Collectively, the individual pathway scores revealed pathway-specific temporal features of pyroptosis, necroptosis, and ferroptosis after SCI, whereas the composite indices provided complementary evidence for the coordinated activation of an integrated lytic cell death-related transcriptional state. These results support the added value of retaining both individual pathway scores and integrated LCD-related indices for subsequent network-level analyses.

### 3.2 WGCNA identified key co-expression modules significantly associated with sLCDI

**Figure 2:**
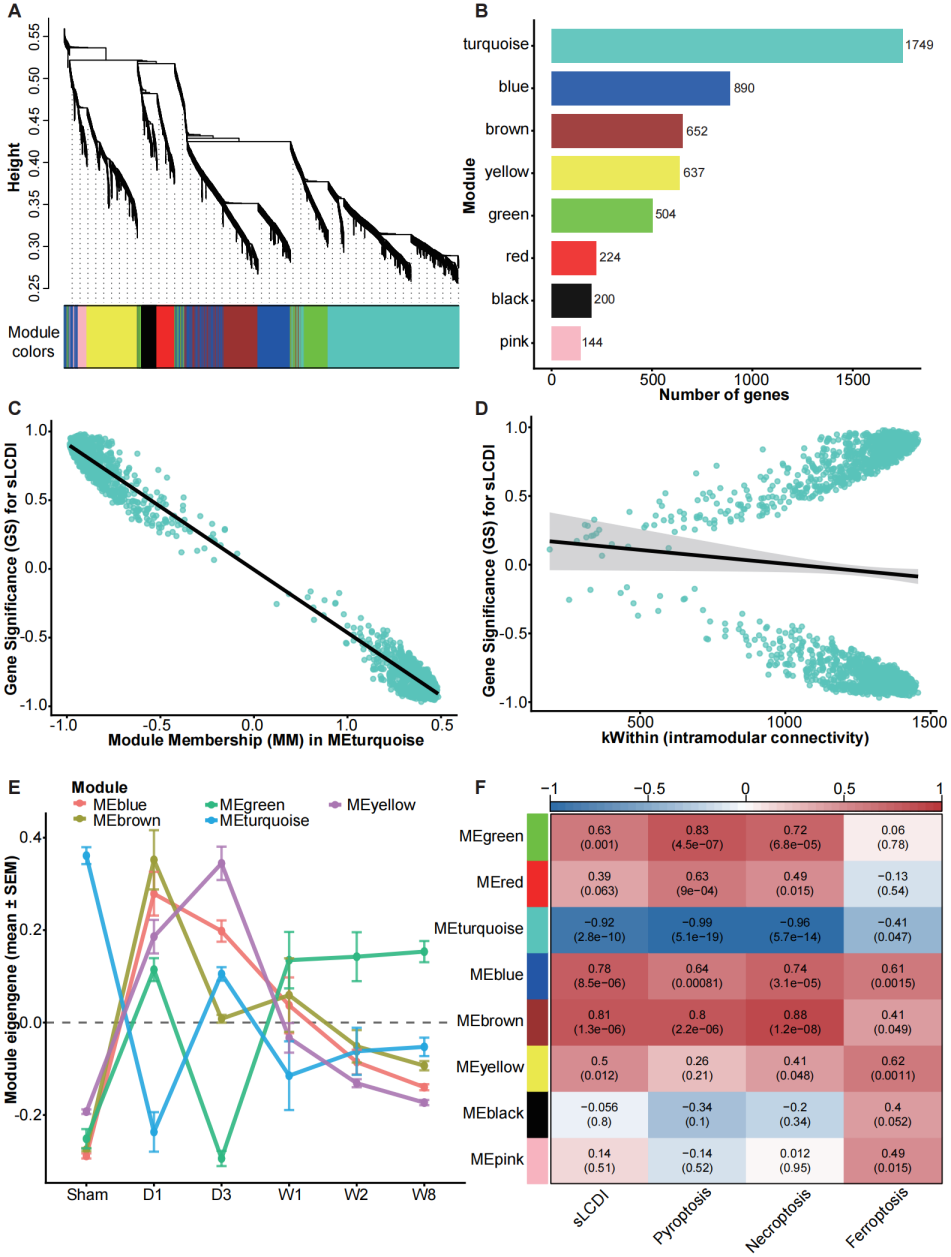
WGCNA identified key co-expression modules associated with lytic cell death activity. All analyses were performed using the GSE45006 dataset. Module–trait correlations were calculated between module eigengenes and lytic cell death-related scores, and the corresponding correlation coefficients and P values are shown in the heatmap. Modules were prioritized according to the absolute strength of their correlation with sLCDI, and the directional interpretation of genes within MEturquoise was further examined in **Supplementary Figure 2**. **(A)** Gene clustering dendrogram and module assignment identified by weighted gene co-expression network analysis (WGCNA). **(B)** Bar plot showing the number of genes in each co-expression module. **(C)** Scatter plot showing the correlation between module membership (MM) and gene significance (GS) for the Standardized LCD Index (sLCDI) in the turquoise module. **(D)** Scatter plot showing the relationship between intramodular connectivity (kWithin) and GS for sLCDI in the turquoise module. **(E)** Dynamic changes in the eigengenes of the major modules across different time points after spinal cord injury. **(F)** Heatmap showing the correlations of module eigengenes with sLCDI and the ssGSEA scores of pyroptosis, necroptosis, and ferroptosis.

WGCNA identified a total of eight co-expression modules, and the hierarchical clustering dendrogram together with module color assignment is shown in **Figure 2A**. Among these modules, the turquoise module contained the largest number of genes (**Figure 2B**). Within the turquoise module, module membership (MM) showed a clear relationship with gene significance (GS) for sLCDI (**Figure 2C**), supporting the relevance of this module to the lytic cell death-related transcriptional state. Intramodular connectivity (kWithin) displayed an overall negative trend with GS for sLCDI (**Figure 2D**), indicating that genes within the turquoise module showed heterogeneous directional relationships between network connectivity and sLCDI association.

Dynamic analysis of module eigengenes showed that MEturquoise was relatively highly expressed in the Sham group but was generally downregulated after injury, whereas several other modules exhibited transient increases during the early post-injury stage (**Figure 2E**). Further module–trait correlation analysis demonstrated that MEturquoise showed the strongest absolute correlation with sLCDI, although the direction of correlation was negative (r = -0.92, P = 2.8 × 10^-10^). MEturquoise was also significantly negatively correlated with pyroptosis, necroptosis, and ferroptosis scores. By contrast, MEblue and MEbrown were positively correlated with sLCDI and multiple lytic cell death-related features, among which MEbrown showed the strongest positive correlation with necroptosis (r = 0.88, P = 1.2 × 10^-8^) (**Figure 2F**).

Because WGCNA modules are defined by gene co-expression topology rather than by the direction of association with external traits, a negatively correlated module may still contain genes that are positively associated with sLCDI. We therefore further examined the directional structure of MEturquoise. As shown in **Supplementary Figure 2**, Cd14 was significantly upregulated at all post-injury time points compared with the Sham group (**Supplementary Figure 2A**). Although Cd14 was negatively correlated with MEturquoise (MM = -0.816), it was strongly positively correlated with sLCDI (GS = 0.940) (**Supplementary Figure 2B**). Directional subcomponent analysis showed that the 1,749 genes in the turquoise module could be divided into 845 sLCDI-activated anti-eigengene genes and 904 sLCDI-suppressed eigengene-aligned genes (**Supplementary Figure 2C**). Cd14 was located within the sLCDI-activated anti-eigengene component (**Supplementary Figure 2D**). Therefore, MEturquoise was selected because it had the strongest overall association with sLCDI, while Cd14 was prioritized as a positively sLCDI-associated anti-eigengene gene within this negatively correlated module.

### 3.3 sLCDI-related candidate genes are mainly enriched in inflammatory immune activation and lytic cell death-related pathways

**Figure 3:**
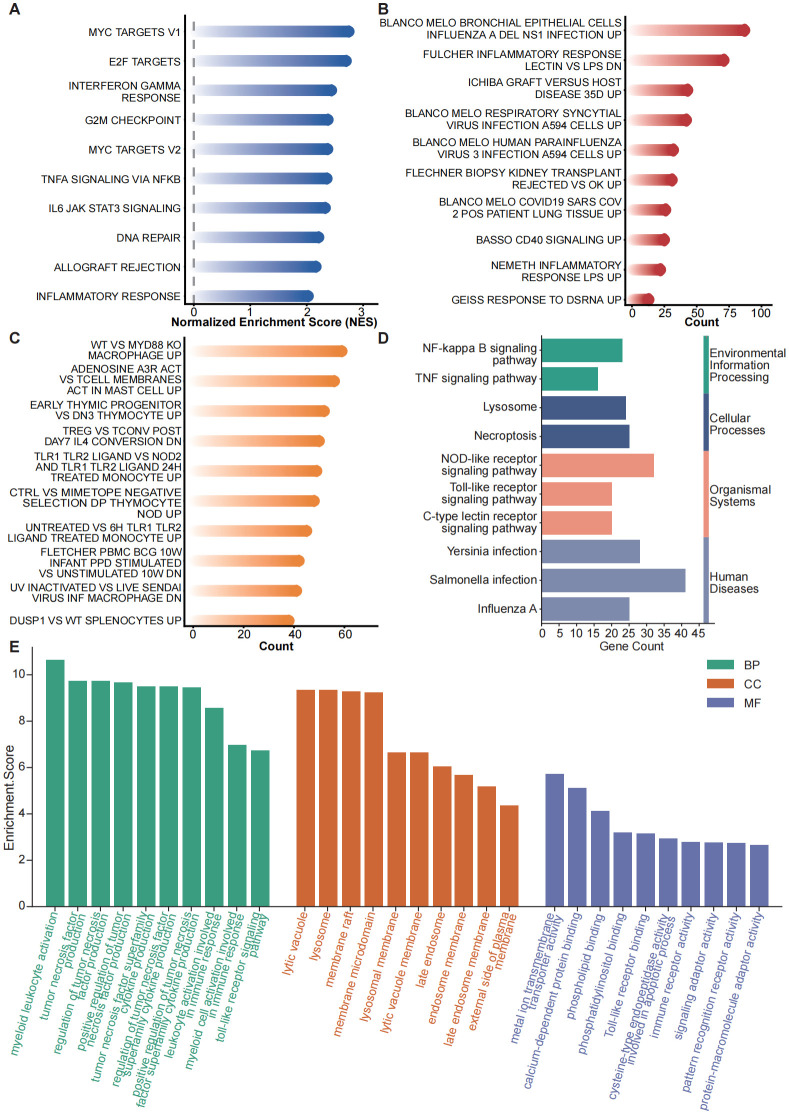
Functional enrichment analysis of sLCDI-related candidate genes. **(A)** Significantly enriched Hallmark pathways of the sLCDI-related candidate gene set. Enrichment analyses were performed using the LCD-related candidate gene set. P values were adjusted using the Benjamini–Hochberg FDR method where applicable, and representative significantly enriched terms are shown. **(B)** Significantly enriched DrugPerturb terms of the sLCDI-related candidate gene set. **(C)** Significantly enriched ImmuneSig terms of the sLCDI-related candidate gene set. **(D)** Kyoto Encyclopedia of Genes and Genomes (KEGG) pathway enrichment analysis of the sLCDI-related candidate gene set. **(E)** Gene Ontology (GO) enrichment analysis showing representative terms for biological process (BP), cellular component (CC), and molecular function (MF).

After GO, KEGG, and MSigDB enrichment analyses of the sLCDI-related candidate gene set, the most significantly enriched Hallmark pathways included MYC targets, E2F targets, interferon-γ response, G2M checkpoint, TNFα signaling via NF-κB, IL6–JAK–STAT3 signaling, and inflammatory response (**Figure 3A**). These findings suggest that the candidate genes are closely associated with cell cycle regulation, inflammatory activation, and immune responses.

Further analysis showed that DrugPerturb enrichment was mainly related to transcriptional responses associated with infection and inflammation, including signatures linked to viral infection, LPS stimulation, CD40 signaling, and graft-versus-host disease-like responses (**Figure 3B**). Meanwhile, ImmuneSig enrichment was primarily concentrated in immune programs related to myeloid/macrophage activation, TLR ligand stimulation, and pathogen-responsive pathways (**Figure 3C**), further supporting an important role for innate immune activation in the molecular network associated with sLCDI.

KEGG analysis demonstrated that these candidate genes were significantly enriched in the NF-κB, TNF, NOD-like receptor, Toll-like receptor, C-type lectin receptor, and necroptosis pathways, as well as in multiple infection-related pathways (**Figure 3D**). GO analysis further showed that these genes were mainly involved in biological processes such as myeloid leukocyte activation and the regulation of cytokine and TNF production, were primarily localized to cellular components related to the lysosome, lytic vacuole, and membrane raft, and were associated with molecular functions such as receptor–ligand activity (**Figure 3E**). Collectively, these findings suggest that sLCDI-related candidate genes may contribute to the secondary pathological progression after SCI through coordinated regulation of innate immune-inflammatory networks and lytic cell death-related signaling pathways.

### 3.4 Integrated multi-machine learning analysis identified *Cd14* as the sLCDI-related hub gene

**Figure 4:**
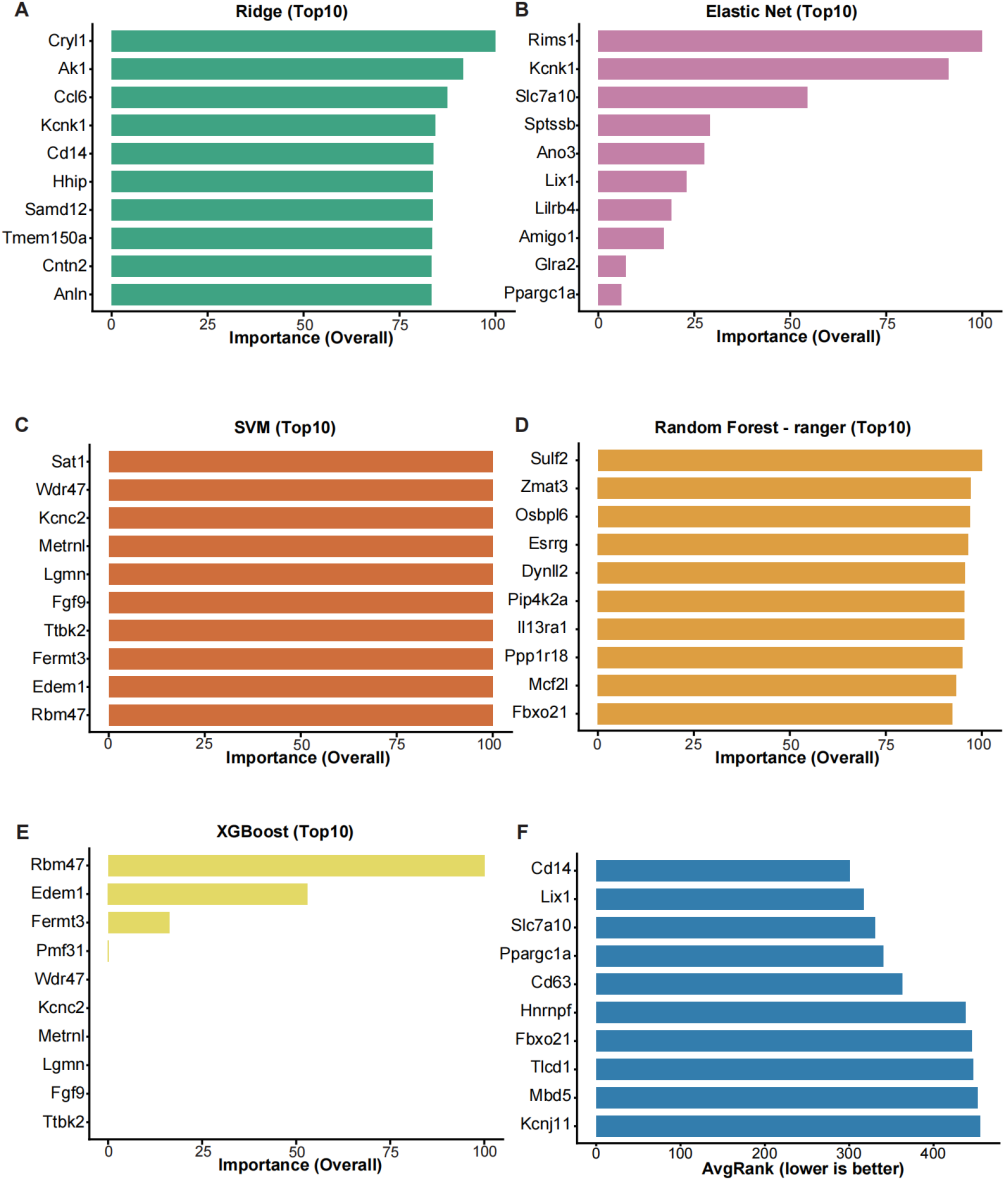
Prioritization of sLCDI-related candidate genes using multiple machine learning models. Feature selection was performed using the GSE45006 dataset, with Sham samples as controls and all post-injury samples combined as the Injury group. Gene importance was evaluated using repeated cross-validation, and the integrated average rank (AvgRank) was used for consensus prioritization. **(A)** Top 10 genes identified by the Ridge model. **(B)** Top 10 genes identified by the Elastic Net model. **(C)** Top 10 genes identified by the support vector machine (SVM) model. **(D)** Top 10 genes identified by the random forest-ranger model. **(E)** Top 10 genes identified by the XGBoost model. **(F)** Top 10 candidate genes identified by integrating the average rankings (AvgRank) across the five models.

Feature selection using five independent machine learning algorithms yielded partially overlapping but distinct sets of top-ranked genes (**Figures 4A–E**). The Ridge model identified *Cryl1*, *Ak1*, *Ccl6*, *Kcnk1*, and *Cd14* among the most important genes, whereas the Elastic Net model prioritized *Rims1*, *Kcnk1*, *Slc7a10*, *Lix1*, and *Ppargc1a*. The SVM model highlighted *Sat1*, *Wdr47*, *Kcnc2*, *Metrnl*, *Lgmn*, and *Fgf9*, while the random forest-ranger model preferentially selected *Sulf2*, *Zmat3*, *Osbpl6*, *Esrrg*, *Dynll2*, and *Fbxo21*. In the XGBoost model, *Rbm47*, *Edem1*, and *Fermt3* ranked highest.Despite differences among individual algorithms, integration of all five models produced a relatively stable consensus ranking. Based on AvgRank, *Cd14* showed the best overall performance, followed by *Lix1*, *Slc7a10*, *Ppargc1a*, and *Cd63* (**Figure 4F**). These results indicate that *Cd14* was the most robust and discriminative gene associated with sLCDI across the multi-model framework and was therefore selected as the hub gene for subsequent validation.

### 3.5 The top 10 candidate genes exhibited distinct expression patterns in SCI

**Figure 5:**
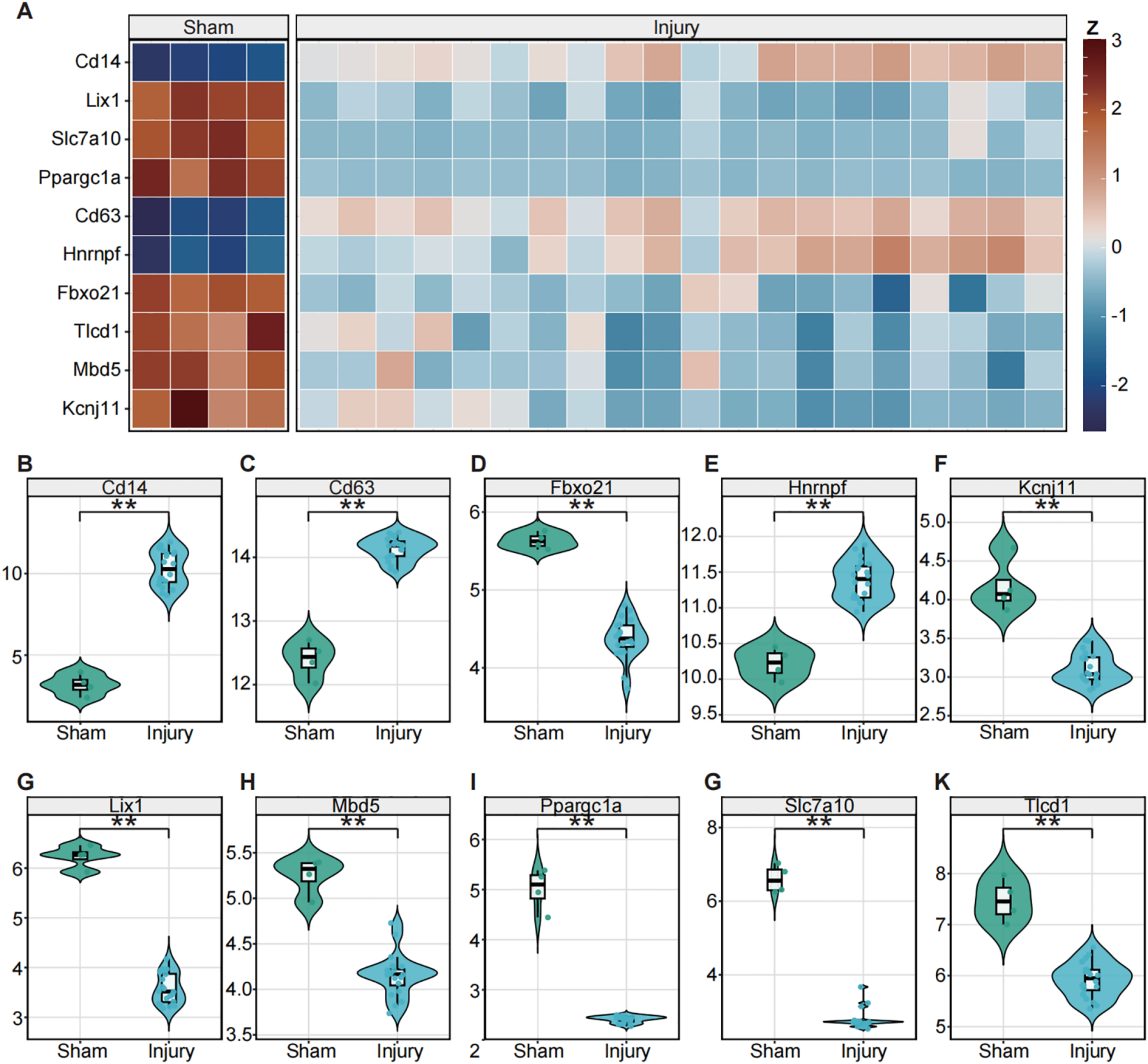
Expression patterns of candidate genes identified by integrated machine learning in the Sham and Injury groups. The GSE45006 dataset included 4 Sham samples and 20 Injury samples. Statistical comparisons between the Sham and Injury groups were performed using the Wilcoxon rank-sum test, followed by Benjamini–Hochberg FDR correction across the 10 candidate-gene comparisons. **FDR < 0.01. **(A)** Heatmap showing the expression patterns of the top 10 candidate genes in the Sham and Injury groups. **(B–K)** Violin plots showing the differential expression of the candidate genes, including Cd14 **(B)**, Cd63 **(C)**, Fbxo21 **(D)**, Hnrnpf **(E)**, Kcnj11 **(F)**, Lix1 **(G)**, Mbd5 **(H)**, Ppargc1a **(I)**, Slc7a10 **(J)**, and Tlcd1 **(K)**, between the two groups.

The heatmap showed that the top 10 candidate genes identified by the integrated multi-machine learning analysis effectively distinguished the Sham group from the Injury group, with a clear separation in overall expression patterns (**Figure 5A**). Among these genes, *Cd14* was markedly upregulated in the Injury group (**Figure 5B**), and *Cd63* was also increased (**Figure 5C**), whereas *Fbxo21* was downregulated after injury (**Figure 5D**). In addition, *Hnrnpf* was elevated in the Injury group (**Figure 5E**), while *Kcnj11* was markedly decreased (**Figure 5F**). The remaining candidate genes, including *Lix1*, *Mbd5*, *Ppargc1a*, *Slc7a10*, and *Tlcd1*, were all significantly downregulated in the Injury group compared with the Sham group (**Figures 5G–K**).

Single-gene violin plots further confirmed that all 10 genes were significantly differentially expressed between the two groups after Benjamini–Hochberg FDR correction (all FDR < 0.01). Notably, *Cd14* showed the most prominent increase, which, together with its top performance in the integrated ranking analysis, further supports its role as a key hub gene associated with sLCDI in SCI.

### 3.6 *Cd14* was positively associated with lytic cell death-related programs and increased dynamically after injury

**Figure 6:**
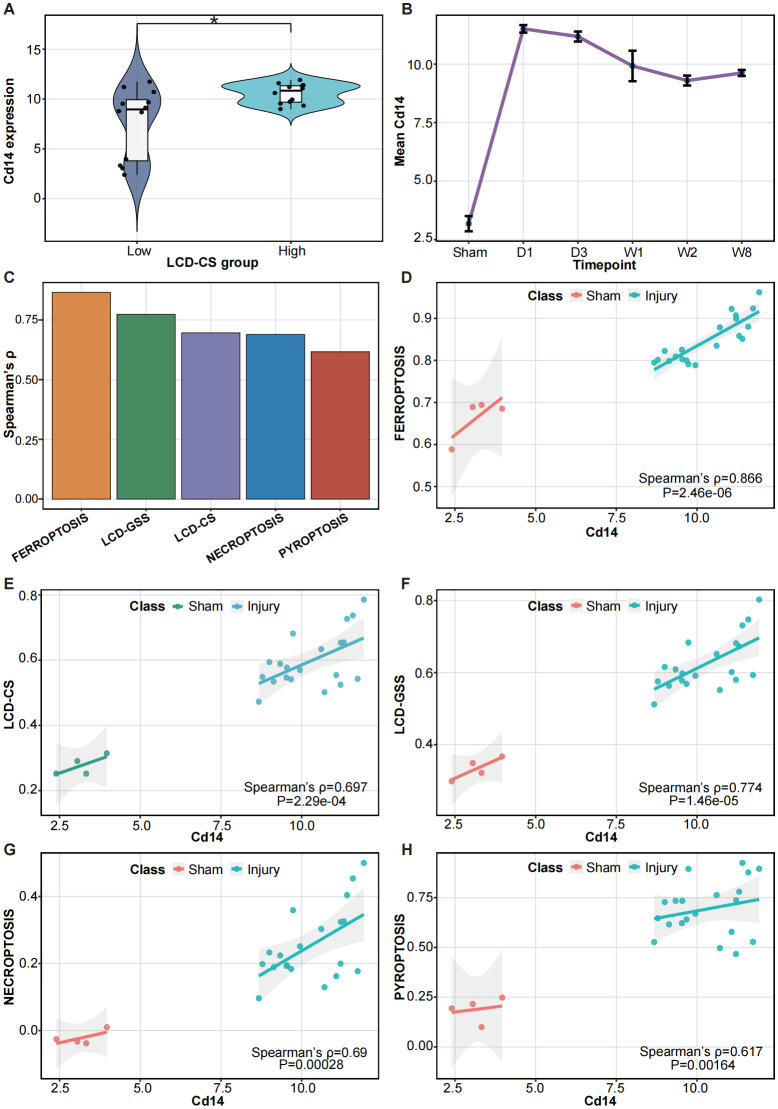
Association of *Cd14* with lytic cell death-related features after spinal cord injury. All analyses were performed using the GSE45006 dataset. The low-LCD-CS and high-LCD-CS groups were defined according to the median LCD-CS value. Group comparisons were performed using the Wilcoxon rank-sum test, and correlations were assessed using Spearman correlation analysis. Spearman’s ρ and P values are shown in the corresponding panels. **(A)** Comparison of *Cd14* expression between the low-LCD-CS and high-LCD-CS groups stratified by the median LCD-CS. **(B)** Dynamic changes in the mean expression of *Cd14* at different time points after injury. **(C)** Summary of Spearman correlation coefficients between Cd14 expression and LCD-related transcriptional scores. **(D–H)** Scatterplots showing the associations between Cd14 expression and individual or composite LCD-related scores.

After stratification according to the median LCD-CS, *Cd14* expression was significantly higher in the high-LCD-CS group than in the low-LCD-CS group (**Figure 6A**). Temporal analysis showed that Cd14 was markedly upregulated as early as 1 day after injury, reached a high level during the acute phase, and remained consistently elevated from D3 to W8 compared with the Sham group (**Figure 6B**). Summary correlation analysis showed that Cd14 expression was positively associated with the overall panel of lytic cell death-related transcriptional scores, including individual pathway scores and composite LCD-related indices (**Figure 6C**). Scatterplot analyses further confirmed consistent positive associations between Cd14 expression and these LCD-related scores (**Figures 6D–H**). Collectively, these findings indicate that increased Cd14 expression is closely associated with sustained activation of lytic cell death-related transcriptional programs after SCI.

### 3.7 The GSE151371 validation cohort further supported the association between CD14 and lytic cell death activation

**Figure 7:**
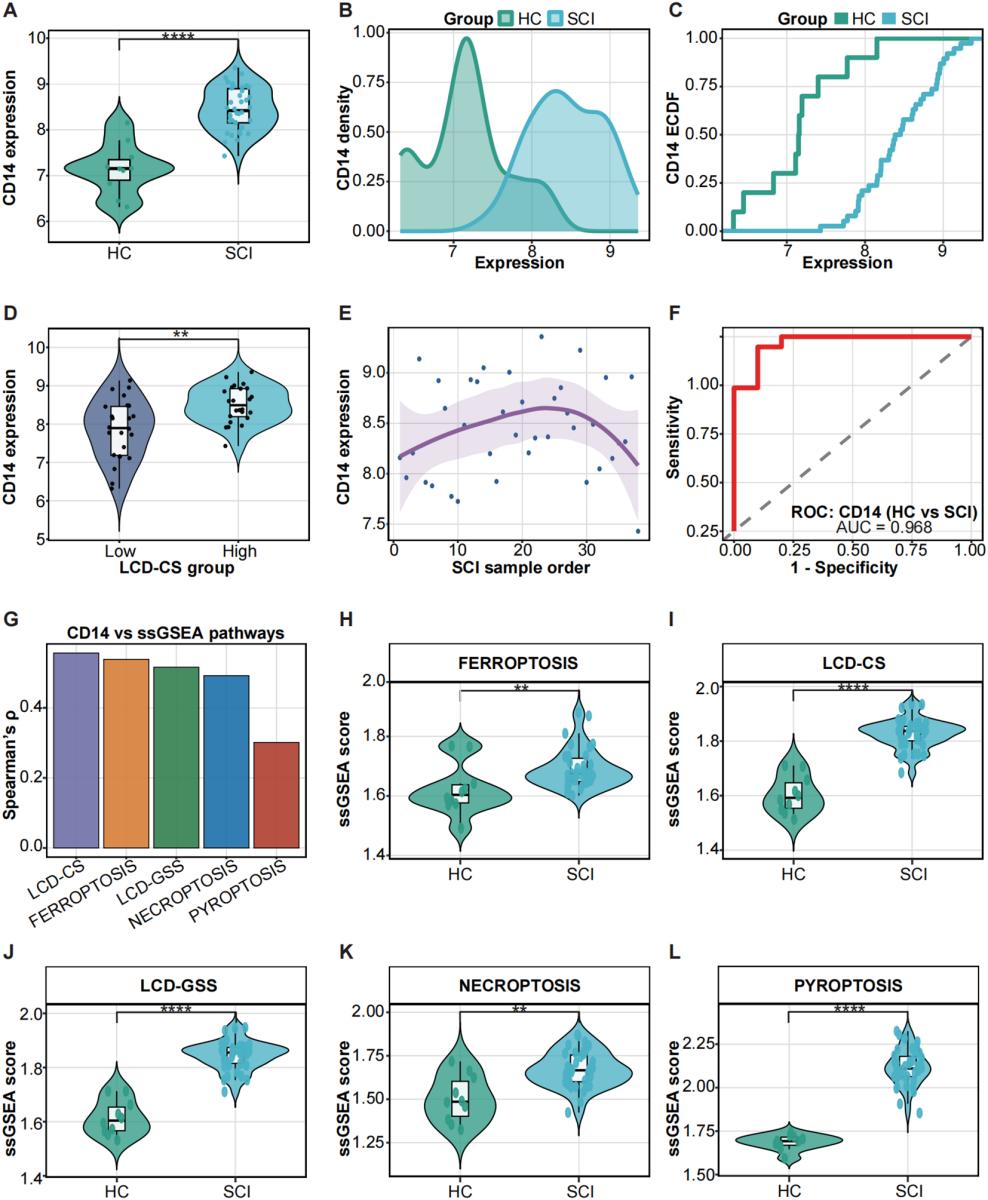
Validation analysis of CD14 and lytic cell death-related transcriptional signatures in the human GSE151371 peripheral white blood cell RNA-seq cohort. GSE151371 is a human peripheral white blood cell RNA-seq dataset. In this analysis, 10 healthy controls and 38 traumatic SCI samples were retained. Group comparisons were performed using the Wilcoxon rank-sum test. Correlations were assessed using Spearman correlation analysis. ROC analysis was used to evaluate the discriminatory ability of CD14. **(A)** Comparison of CD14 expression between the healthy control (HC) and spinal cord injury (SCI) groups. **(B)** Density distribution plot of CD14 expression in the HC and SCI groups. **(C)** Empirical cumulative distribution function (ECDF) curves of CD14 expression in the HC and SCI groups. **(D)** Comparison of CD14 expression between the low-LCD-CS and high-LCD-CS groups. **(E)** Expression trend of CD14 across SCI samples. **(F)** Receiver operating characteristic (ROC) curve of CD14 for distinguishing SCI from HC. **(G)** Summary of Spearman correlation coefficients between CD14 expression and LCD-related transcriptional scores. **(H–L)** Comparisons of individual and composite LCD-related scores between the HC and SCI groups.

In the GSE151371 validation cohort, CD14 expression was significantly higher in the SCI group than in the HC group (**Figure 7A**). Density distribution and empirical cumulative distribution function (ECDF) curves further showed an overall shift of CD14 expression toward higher levels in the SCI group, with a clear separation trend from the HC group (**Figures 7B, C**). After stratification according to the median LCD-CS, CD14 expression was also significantly higher in the high-LCD-CS group than in the low-LCD-CS group (**Figure 7D**). Within the SCI samples, CD14 expression remained consistently high with only mild fluctuation, suggesting a relatively stable upregulated pattern in the external cohort (**Figure 7E**). ROC analysis showed that CD14 had strong discriminatory ability for distinguishing SCI from HC, with an AUC of 0.968 (**Figure 7F**).

Correlation analysis in GSE151371 showed a consistent positive association between CD14 expression and LCD-related transcriptional scores (**Figure 7G**). In parallel, multiple LCD-related scores were higher in the SCI group than in the HC group (**Figures 7H–L**). Collectively, these results support the upregulation of CD14 and its association with lytic cell death-related transcriptional signatures in a human acute SCI-related peripheral blood dataset. However, because GSE151371 lacks fine-grained post-injury time-point information, it should not be interpreted as validation of the detailed temporal trajectory observed in the discovery cohort.

### 3.8 Immunofluorescence analysis reveals spatial associations between CD14 and lytic cell death-related markers after spinal cord injury

**Figure 8:**
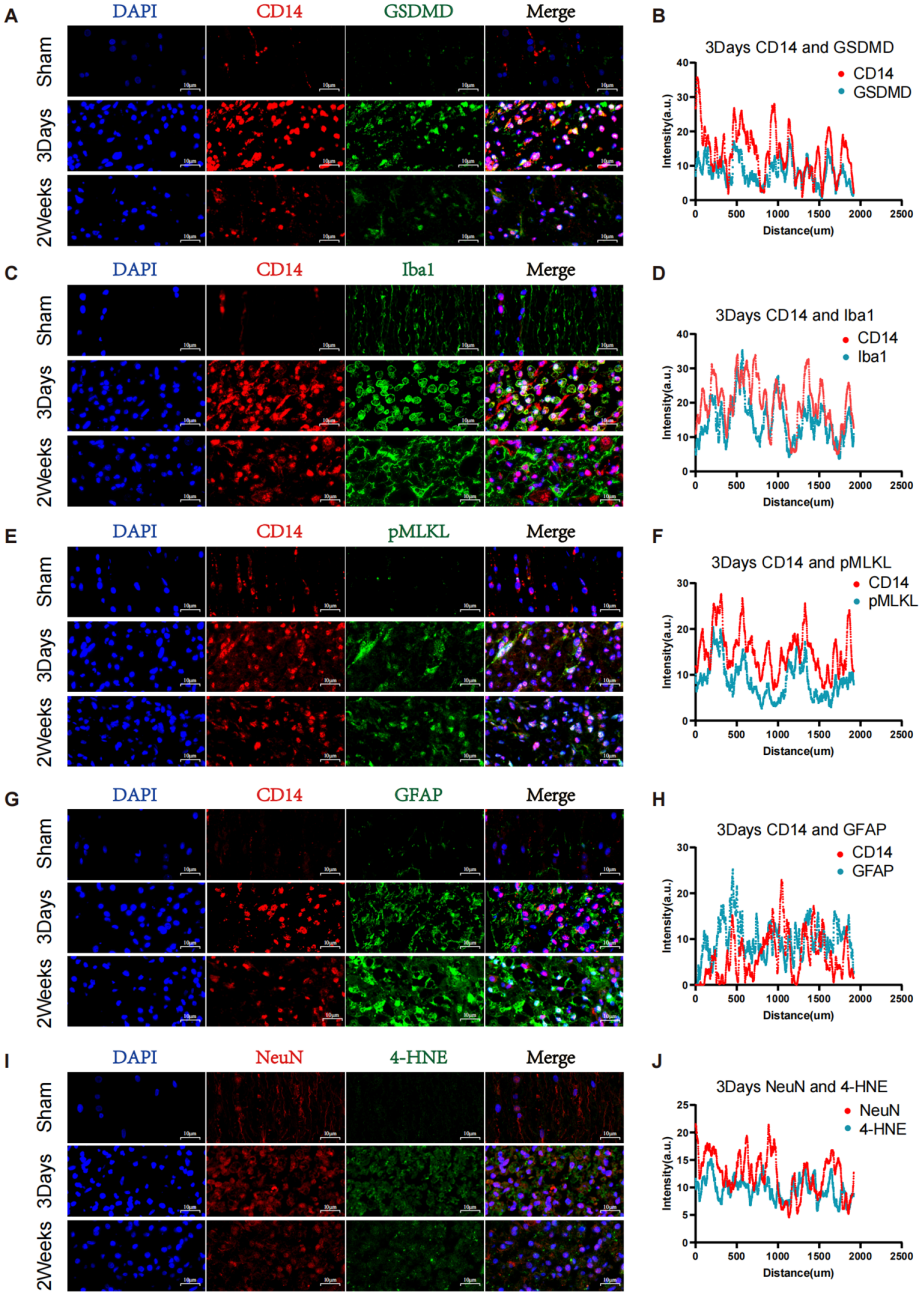
Immunofluorescence analysis of CD14 and lytic cell death-related markers after spinal cord injury. Representative images are shown from rat spinal cord tissues in the Sham group and at 3 days and 2 weeks after SCI. Scale bars are indicated in the images. Co-localization profiles represent fluorescence intensity changes along the selected regions. These images were used to show spatial association rather than causal regulation. Representative images are shown from n = 6 rats per group. **(A, B)** Representative immunofluorescence images of CD14 and GSDMD in spinal cord tissues from the Sham group and at 3 days and 2 weeks after injury, together with the corresponding co-localization profiles. **(C, D)** Representative images of CD14 and Iba1, together with the corresponding co-localization profiles. **(E, F)** Representative images of CD14 and p-MLKL, together with the corresponding co-localization profiles. **(G, H)** representative images of CD14 and GFAP showing their spatial distribution, together with corresponding fluorescence intensity profiles. **(I, J)** Representative images of NeuN and 4-HNE, together with the corresponding co-localization profiles.

To further examine the histological association between CD14 and lytic cell death-related markers after SCI, double immunofluorescence staining was performed in spinal cord tissues from the Sham group and at 3 days and 2 weeks after injury. To improve the visualization and interpretation of the staining patterns, representative whole-lesion overview images together with corresponding higher-magnification regions were provided in **Supplementary Figure 3**. In the Sham group, CD14 and GSDMD showed relatively weak immunofluorescence signals. After SCI, both CD14 and GSDMD signals increased markedly at 3 days and remained detectable at 2 weeks. The merged images and corresponding co-localization profiles showed spatial overlap between CD14-positive signals and GSDMD-positive regions, particularly during the acute phase after injury (**Figures 8A, B**; **Supplementary Figures 4A, B**, **5F**). These findings suggest that CD14-positive cells are spatially associated with pyroptosis-related molecular signals in the injured spinal cord.

Similarly, CD14 and Iba1 signals were both increased after SCI. At 3 days after injury, CD14-positive signals were closely distributed within Iba1-positive regions, consistent with the enrichment of CD14 in activated microglia/macrophage-related populations (**Figures 8C, D**; **Supplementary Figures 4C, D**, **5G**). At 2 weeks, Iba1 immunoreactivity remained evident, although the spatial overlap between CD14 and Iba1 appeared less prominent than that observed at 3 days. These results support an association between CD14 expression and myeloid inflammatory activation after SCI. CD14 and p-MLKL also showed increased signals after SCI. Spatial overlap between CD14-positive and p-MLKL-positive signals was observed at both 3 days and 2 weeks after injury (**Figures 8E, F**; **Supplementary Figures 4E, F**, **5H**), suggesting that CD14-positive regions are associated with necroptosis-related molecular signals during SCI progression.

GFAP immunoreactivity increased after SCI and became more prominent at 2 weeks, consistent with reactive astrocytic responses in the injured spinal cord. At this time point, CD14-positive signals were located adjacent to or partially overlapped with GFAP-positive regions (**Figures 8G, H**; **Supplementary Figures 4G, H**, **5I**). However, this spatial relationship should not be interpreted as direct CD14 expression by astrocytes. In light of the single-cell reanalysis showing that Cd14 is mainly enriched in myeloid populations whereas Gfap is predominantly enriched in astrocytes, the CD14/GFAP staining pattern is more appropriately interpreted as a spatial association between CD14-positive myeloid cells and GFAP-positive reactive astrocytic regions within the injured spinal cord microenvironment.

Regarding neuronal lipid peroxidation-related injury, 4-HNE immunoreactivity increased after SCI and remained detectable at 2 weeks, indicating persistent lipid peroxidation-related stress in the injured tissue (**Figures 8I, J**; **Supplementary Figures 4I, J**, **5J**). The NeuN/4-HNE staining further suggested that oxidative lipid damage was present in the neuronal microenvironment after injury. Collectively, these immunofluorescence findings indicate that CD14-positive signals are spatially associated with myeloid inflammatory activation and lytic cell death-related molecular signals after SCI. Together with the RT-qPCR and single-cell reanalysis results, these data support an association between CD14-positive myeloid inflammatory responses and lytic cell death-related signatures in the injured spinal cord, while not establishing a direct causal or cell-type-specific regulatory role for CD14.

### 3.9 RT-qPCR further supports *Cd14* as a core gene associated with lytic cell death after SCI

**Figure 9:**
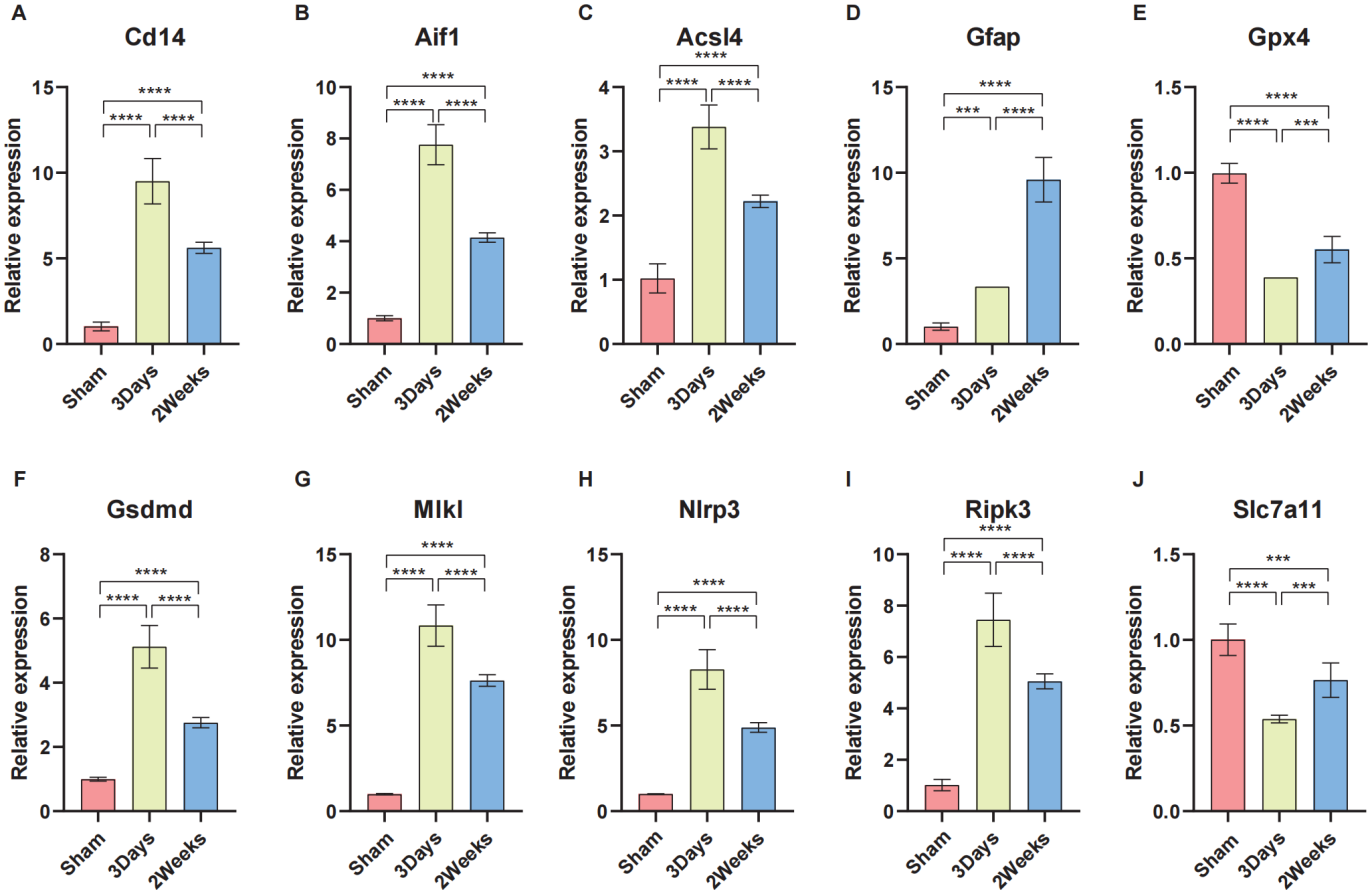
RT-qPCR validation of *Cd14* and lytic cell death-related genes after spinal cord injury. Data are presented as mean ± SD. Statistical comparisons among the Sham, 3-day, and 2-week groups were performed using one-way ANOVA. *P < 0.05, **P < 0.01, ***P < 0.001, ****P < 0.0001. n = 6 biological replicates per group. **(A–J)** Relative mRNA expression levels of *Cd14* **(A)**, *Aif1* **(B)**, *Acsl4* **(C)**, *Gfap* **(D)**, *Gpx4* **(E)**, *Gsdmd* **(F)**, *Mlkl* **(G)**, *Nlrp3* **(H)**, *Ripk3* **(I)**, and *Slc7a11* **(J)** in spinal cord tissues from the Sham group and at 3 days and 2 weeks after injury.

RT-qPCR analysis showed that *Cd14* was significantly upregulated at 3 days after SCI and remained elevated at 2 weeks, although its expression was slightly reduced compared with the 3-day time point (**Figure 9A**). The microglia/macrophage marker *Aif1* showed a similar pattern, reaching its highest level at 3 days and remaining significantly higher than that in the Sham group at 2 weeks (**Figure 9B**). The ferroptosis-related gene *Acsl4* was also markedly increased after injury, with the most pronounced elevation observed at 3 days, followed by a partial decline at 2 weeks (**Figure 9C**). In contrast, *Gfap* showed a sustained increase over time and reached its highest level at 2 weeks, suggesting a progressive reactive astrocytic response during the subacute injury stage. (**Figure 9D**).

Among the anti-ferroptotic genes, *Gpx4* was significantly downregulated after SCI and remained suppressed at 2 weeks (**Figure 9E**). The pyroptosis-related gene *Gsdmd* was markedly upregulated at 3 days and remained elevated at 2 weeks (**Figure 9F**). Similarly, the necroptosis- and inflammation-related genes *Mlkl*, *Nlrp3*, and *Ripk3* were all significantly increased after SCI, although their expression levels showed a partial decline from 3 days to 2 weeks (**Figures 9G–I**). By contrast, *Slc7a11* was significantly decreased after SCI and showed only partial recovery at 2 weeks (**Figure 9J**).

The supplementary heatmap further showed a clear overall separation among the Sham, 3-day, and 2-week groups (**Supplementary Figure 6A**). Temporal trend analysis likewise indicated that *Cd14* and several lytic cell death-related genes shared a similar pattern characterized by early induction followed by partial decline, whereas *Gfap* showed delayed upregulation and *Gpx4/Slc7a11* remained persistently suppressed after injury (**Supplementary Figure 6B**). Collectively, these findings further support a close association between *Cd14* and lytic cell death-related pathways during SCI progression.

### 3.10 Single-cell analysis

**Figure 10:**
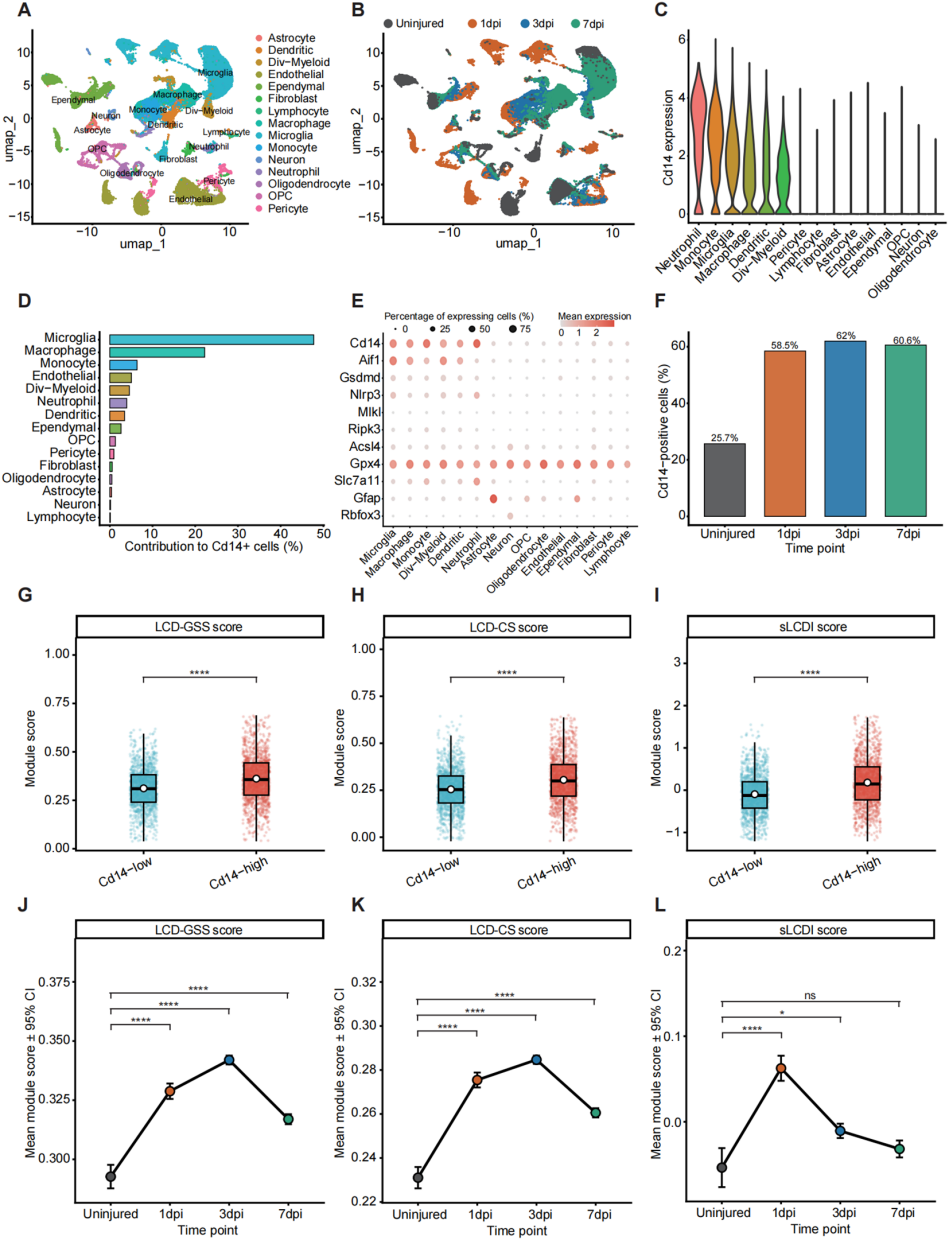
Single-cell analysis of GSE162610 reveals the cellular sources of *Cd14*-positive cells and their association with LCD-related transcriptional states in myeloid cells. GSE162610 included uninjured spinal cord and 1, 3, and 7 dpi SCI samples. **(A)** UMAP plot of the GSE162610 single-cell dataset showing the distribution of major spinal cord cell types. **(B)** UMAP plot showing the distribution of cells from different injury time points, including uninjured spinal cord and 1, 3, and 7 days post-injury (dpi). **(C)** Expression distribution of *Cd14* across different cell types. **(D)** Cellular composition of all *Cd14*-positive cells. **(E)** Cell-type distribution of RT-qPCR/immunofluorescence-related marker genes. Dot size represents the percentage of cells expressing each gene, and color intensity indicates the average expression level. **(F)** Changes in the proportion of *Cd14*-positive cells across different injury time points. **(G–I)** Comparison of LCD-GSS, LCD-CS, and sLCDI scores between *Cd14*-low and *Cd14*-high myeloid cells. For visualization, the y-axis was restricted to the 1st–99th percentile range. **(J–L)** Mean sLCDI, LCD-GSS, and LCD-CS scores with 95% confidence intervals across different injury time points in myeloid cells. Statistical comparisons were performed using the Wilcoxon rank-sum test followed by Benjamini–Hochberg FDR correction. *P < 0.05, ****P < 0.0001; ns, not significant.

UMAP analysis showed that the GSE162610 dataset contained diverse spinal cord cell populations, and cells from different injury time points displayed distinct distribution patterns in the UMAP space (**Figures 10A, B**). *Cd14* was highly expressed in neutrophils, monocytes, microglia, macrophages, dendritic cells, and dividing myeloid cells (**Figure 10C**). Further analysis of the cellular sources of all *Cd14*-positive cells revealed that microglia and macrophages were the major contributors (**Figure 10D**). Analysis of RT-qPCR/immunofluorescence-related marker genes showed that *Cd14* and *Aif1* were mainly enriched in myeloid cells, *Gfap* was predominantly enriched in astrocytes, and *Rbfox3* was mainly enriched in neurons. Several LCD-related marker genes, including *Gsdmd*, *Nlrp3*, *Ripk3*, and *Slc7a11*, also showed detectable expression in myeloid populations (**Figure 10E**). Across injury time points, the proportion of *Cd14*-positive cells markedly increased at 1 dpi and remained at relatively high levels at 3 and 7 dpi (**Figure 10F**).

We next compared LCD-related scores between *Cd14*-low and *Cd14*-high myeloid cells. *Cd14*-high myeloid cells exhibited significantly higher LCD-GSS, LCD-CS, and sLCDI scores than *Cd14*-low cells, suggesting that *Cd14*-high myeloid cells were associated with a stronger LCD-related transcriptional state (**Figures 10G–I**). In addition, integrated LCD-related scores in myeloid cells showed dynamic changes after SCI. LCD-GSS and LCD-CS increased after injury, indicating activation of LCD-related transcriptional programs in myeloid cells following SCI (**Figures 10J–L**). **Supplementary Figure 7** further illustrates the UMAP expression patterns of 11 RT-qPCR/immunofluorescence-related marker genes at the single-cell level.

## 4 Discussion

Secondary SCI is driven by a temporally organized pathological cascade involving immune activation, oxidative stress, blood-spinal cord barrier disruption, glial reactivity, and progressive cellular loss, all of which interact to expand tissue damage beyond the original lesion core (6, 36–38). In this study, we integrated transcriptomic scoring, co-expression network analysis, machine learning-based prioritization, external human validation, rat model validation, and single-cell reanalysis to characterize lytic cell death-related transcriptional programs after SCI. The main contribution of this work is not the confirmation of any single cell death pathway, but the identification of an integrated lytic cell death-related transcriptional state that is closely associated with immune-inflammatory activation and myeloid cell responses after injury. In this context, the individual pathway scores were used to preserve pathway-specific information, whereas the composite indices were used to summarize the shared and coordinated transcriptional features of pyroptosis, necroptosis, and ferroptosis. Therefore, the composite indices should be interpreted as integrative transcriptomic indicators rather than direct measurements of cell death execution. The dynamic increase in pyroptosis-, necroptosis-, and ferroptosis-related transcriptional scores supports the concept that secondary SCI involves coordinated activation of multiple membrane-disruptive and inflammation-amplifying cell death programs. This pattern is biologically consistent with the acute SCI microenvironment, in which inflammatory mediators, oxidative stress, mitochondrial dysfunction, and lipid peroxidation rapidly accumulate after trauma (6, 19, 21, 22, 38–40). Importantly, these pathways should not be viewed as isolated events. Pyroptosis can promote inflammatory mediator release, necroptosis can amplify tissue damage through RIPK3/MLKL-dependent membrane disruption, and ferroptosis reflects oxidative lipid damage and impaired antioxidant defense (19, 21, 22, 39–54). Therefore, the simultaneous elevation of these signatures suggests that lytic cell death may represent an integrated inflammatory-degenerative axis during SCI progression rather than a set of parallel single-pathway responses.

The enrichment results further indicate that lytic cell death-related signatures are embedded within broader immune and stress-response networks. The enrichment of TNFα/NF-κB, IL6-JAK-STAT3, E2F-related transcriptional responses, and lysosome/lytic vacuole-related terms suggests that the LCD-associated gene network may reflect combined inflammatory activation, myeloid cell responses, cell-cycle-related stress, and impaired intracellular clearance after SCI (55–61). In particular, lysosomal dysfunction may be relevant because lysosomal damage has been linked to the accumulation of RIPK1/RIPK3 and enhanced necroptosis, while lysosomal membrane permeabilization has also been implicated in neuronal pyroptosis after SCI (46, 48). Thus, rather than providing independent mechanistic proof for each pathway, these enrichment findings help define the inflammatory and cellular-stress context in which lytic cell death-related transcriptional programs are activated. Within this integrated framework, CD14 emerged as the most robust candidate hub gene associated with the lytic cell death index. This finding is biologically plausible because CD14 is a myeloid pattern-recognition receptor and TLR co-receptor involved in innate immune sensing, cytokine production, and inflammatory amplification (24, 62). Previous studies have also suggested that CD14 is an important organizer of microglial responses to CNS injury-related signals and that CD14 expression is increased after SCI, primarily in microglia/macrophage populations (25, 63). Consistently, our single-cell reanalysis showed that Cd14 was mainly enriched in myeloid populations, including microglia, macrophages, monocytes, neutrophils, dendritic cells, and dividing myeloid cells. Cd14-high myeloid cells also showed higher LCD-related transcriptional scores than Cd14-low cells, supporting an association between CD14-positive myeloid activation and lytic cell death-related transcriptional states.

The immunofluorescence and RT-qPCR results provide additional tissue-level support for this association. At 3 days after injury, CD14-positive signals were spatially associated with Iba1-, GSDMD-, and p-MLKL-positive regions, suggesting that CD14-positive myeloid inflammatory responses overlap with pyroptosis- and necroptosis-related molecular signals in the acute injured microenvironment. At 2 weeks, CD14-positive signals were located adjacent to or partially overlapped with GFAP-positive reactive astrocytic regions. However, based on the single-cell evidence showing that Cd14 is predominantly enriched in myeloid cells whereas Gfap is enriched in astrocytes, this finding should be interpreted as spatial association between CD14-positive myeloid cells and GFAP-positive reactive astrocytic regions, rather than direct CD14 expression by astrocytes. This interpretation is also consistent with the concept that microglia/macrophage populations and astrocytes interact during scar organization and subacute tissue remodeling after SCI (58–60, 64–67).

From a translational perspective, the present findings suggest that targeting a single downstream cell death pathway may be insufficient to fully modulate secondary injury after SCI. The concurrent activation of pyroptosis-, necroptosis-, and ferroptosis-related signatures indicates that these programs may form a coordinated inflammatory-degenerative network. CD14 is noteworthy because it is positioned at the intersection of injury sensing, myeloid activation, and inflammatory amplification (24, 25, 62, 63). Nevertheless, CD14 should currently be regarded as a candidate biomarker and association-based hub gene rather than a confirmed therapeutic target. Moreover, innate immune responses after SCI are not uniformly detrimental, as microglia and infiltrating macrophages may also contribute to debris clearance and tissue repair in a stage-dependent manner (25, 65, 68). Therefore, future therapeutic strategies should focus on stage-specific immune modulation rather than indiscriminate suppression of CD14-related signaling.

Several limitations of this study should be acknowledged. First, although CD14 was consistently identified as the most robust candidate hub gene through integrated transcriptomic analysis, WGCNA, multi-machine-learning prioritization, external validation, RT-qPCR, immunofluorescence, and single-cell reanalysis, the current evidence remains primarily associative. This study did not include CD14-targeted intervention experiments and therefore cannot establish that CD14 directly regulates pyroptosis, necroptosis, or ferroptosis after SCI. Accordingly, the observed relationships should be interpreted as associations between CD14-related myeloid inflammatory activation and lytic cell death-related signatures, rather than as definitive evidence of causal regulation. Future studies using CD14 neutralization, conditional knockout, gene knockdown, or pharmacological inhibition will be necessary to determine whether CD14 directly modulates inflammasome activation, GSDMD-mediated pyroptosis, RIPK3/MLKL-dependent necroptotic signaling, ferroptotic lipid peroxidation, and secondary injury progression after SCI.

Second, the lytic cell death-related scores used in this study were derived from transcriptomic enrichment analyses and were intended to reflect pathway-associated transcriptional activity rather than direct measurements of cell death execution at the cellular level. Therefore, these scores should not be interpreted as quantitative evidence of actual pyroptotic, necroptotic, or ferroptotic events. Third, although the added single-cell reanalysis improved the cellular resolution of this study and suggested that Cd14 was predominantly enriched in myeloid populations, the immunofluorescence validation was performed on paraffin-embedded thin sections, which may limit the visualization of the complete morphology of microglia/macrophages and astrocytes. Therefore, the immunofluorescence results should be interpreted mainly as evidence of spatial association rather than definitive proof of complete cellular morphology or cell-type-specific expression. Future studies using frozen sections, thicker sections, confocal z-stack imaging, spatial transcriptomics, lineage-tracing approaches, and cell-type-specific perturbation experiments are needed to further validate the cellular localization and functional role of CD14-positive cells after SCI.

In addition, the external validation dataset GSE151371 differs from the discovery cohort in species, sample source, and temporal resolution. Specifically, GSE151371 is a human peripheral white blood cell RNA-seq dataset from patients with acute traumatic SCI, whereas GSE45006 is an experimental spinal cord tissue dataset with multiple post-injury time points. Therefore, GSE151371 supports the external clinical relevance of CD14 expression and its association with lytic cell death-related transcriptional signatures in human SCI-related samples, but it cannot independently validate the fine-grained temporal dynamics observed in the discovery cohort. Human injured spinal cord tissue is extremely difficult to obtain, and this represents an inherent limitation of clinical transcriptomic validation in SCI. To partially compensate for this limitation, we incorporated the published single-cell RNA-seq dataset GSE162610, which includes uninjured and 1, 3, and 7 dpi spinal cord injury-site samples, to provide complementary evidence regarding the cellular distribution and early post-injury temporal pattern of Cd14 expression.

Finally, although the transcriptomic discovery dataset covered multiple post-injury time points up to 8 weeks, the experimental validation by immunofluorescence and RT-qPCR was limited to 3 days and 2 weeks after SCI. These two time points were selected to represent the acute and subacute phases, because lytic cell death-related responses are rapidly activated after SCI and the early post-injury period represents a critical window for secondary injury amplification. However, this design does not fully validate the long-term transcriptional dynamics observed in the discovery cohort, particularly during the later chronic stage. Future studies should include additional time points, such as 1 week, 8 weeks, and later chronic stages, to determine whether CD14-associated myeloid activation and lytic cell death-related molecular signatures persist or change during long-term scar maturation and secondary injury remodeling.

In summary, this study suggests that lytic cell death-related transcriptional programs are dynamically activated after SCI and are closely associated with immune-inflammatory signaling networks. CD14 was identified as a candidate hub gene associated with myeloid inflammatory activation and lytic cell death-related signatures. However, the current findings should be interpreted as evidence of association rather than causation. Further CD14-targeted functional studies are required to determine whether CD14 plays a direct regulatory role in lytic cell death pathway activation and secondary injury progression after SCI.

## 5 Conclusion

In this study, transcriptomic analysis revealed significant time-dependent activation of lytic cell death-related programs after spinal cord injury. Through WGCNA, functional enrichment, and integrated multi-machine-learning analyses, CD14 was identified as a candidate hub gene closely associated with the lytic cell death index. External cohort validation further supported the stable upregulation of CD14 in SCI and its positive correlations with pyroptosis-, necroptosis-, and ferroptosis-related enrichment scores. In addition, immunofluorescence, RT-qPCR, and single-cell reanalysis indicated that CD14 was closely associated with myeloid inflammatory activation and lytic cell death-related molecular signatures in the injured spinal cord microenvironment. Collectively, these findings suggest that CD14 may serve as a candidate biomarker and candidate molecular hub associated with myeloid inflammation and lytic cell death-related transcriptional programs after SCI. However, the current study does not establish a causal regulatory role for CD14. Further functional studies using CD14-targeted interventions are required to determine whether CD14 directly modulates pyroptosis-, necroptosis-, and ferroptosis-related pathways and secondary injury progression after SCI.

## Data Availability

The datasets presented in this study can be found in online repositories. The names of the repository/repositories and accession number(s) can be found in the article.
