# Supplementary material for: Dynamic activation of lytic cell death-related programs identifies CD14 as a candidate hub gene associated with secondary injury after spinal cord injury
Source: Front Immunol. 2026 Jul 6;17:1841784. doi: 10.3389/fimmu.2026.1841784 (PMC13382496; doi:10.3389/fimmu.2026.1841784)
Supplement: Supplementary file 1 [file DataSheet1.pdf]

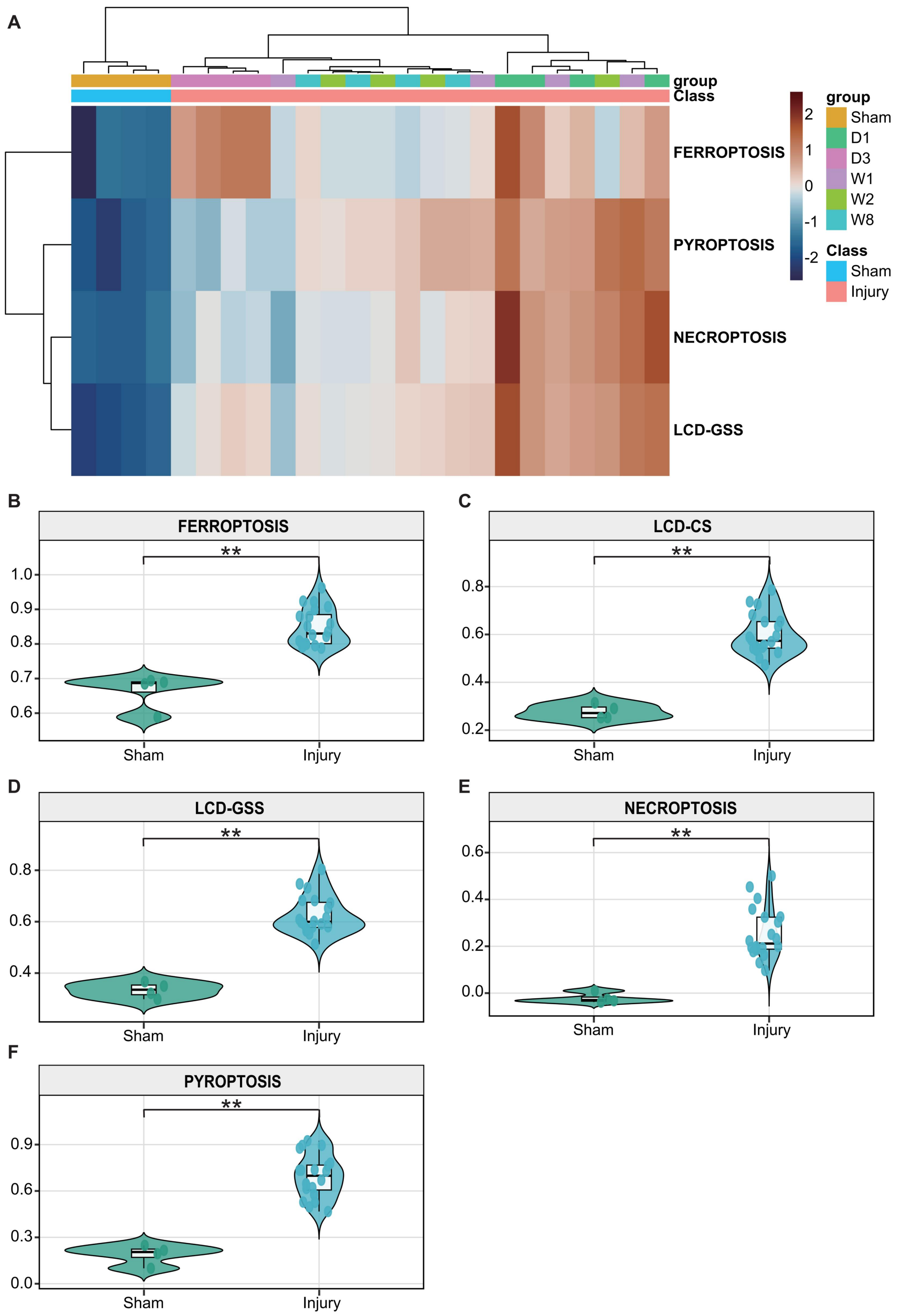

Supplementary Figure 1. Overall increase in lytic cell death-related signatures after spinal cord injury.

(A) Heatmap showing the ssGSEA scores of ferroptosis, pyroptosis, necroptosis, and the LCD Gene Set Score (LCD-GSS) across individual samples in the Sham and Injury groups.

(B–F) Violin plots comparing the ssGSEA scores of ferroptosis (B), LCD Composite Score (LCD-CS) (C), LCD Gene Set Score (LCD-GSS) (D), necroptosis (E), and pyroptosis (F) between the Sham and Injury groups.
**P < 0.01.

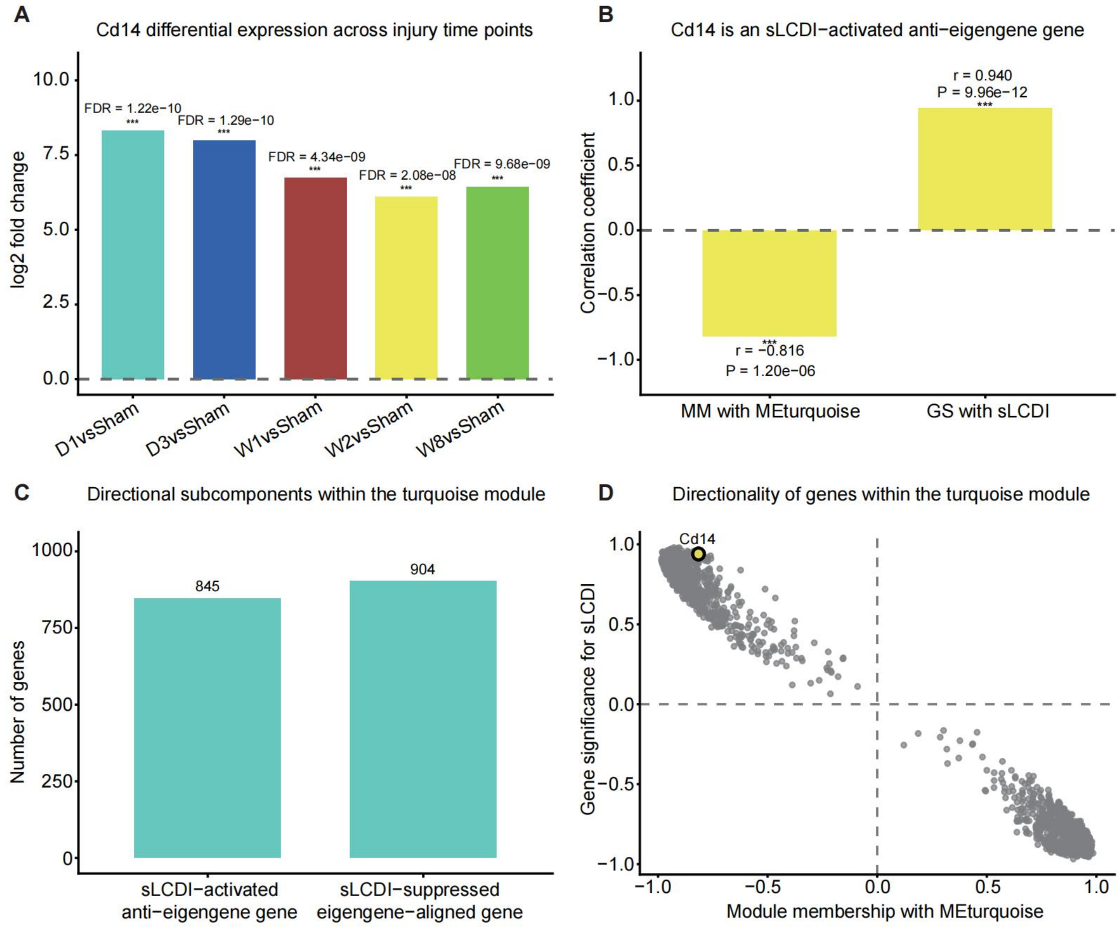

Supplementary Figure 2. Directional analysis of Cd14 within the negatively sLCDI-correlated turquoise module.

(A) Differential expression of Cd14 across post-injury time points compared with the Sham group. Bars show log2 fold changes, and the corresponding FDR values are indicated above each bar.

(B) Correlation pattern of Cd14 with the turquoise module eigengene and sLCDI. Cd14 showed negative module membership with MEturquoise (MM = -0.816) but strong positive gene significance for sLCDI (GS = 0.940).

(C) Directional subcomponents within the turquoise module. The 1,749 genes in this module were divided into 845 sLCDI-activated anti-eigengene genes and 904 sLCDI-suppressed eigengene-aligned genes.

(D) Scatter plot showing the directionality of genes within the turquoise module based on module membership with MEturquoise and gene significance for sLCDI. Cd14 is highlighted and belongs to the sLCDI-activated anti-eigengene component.

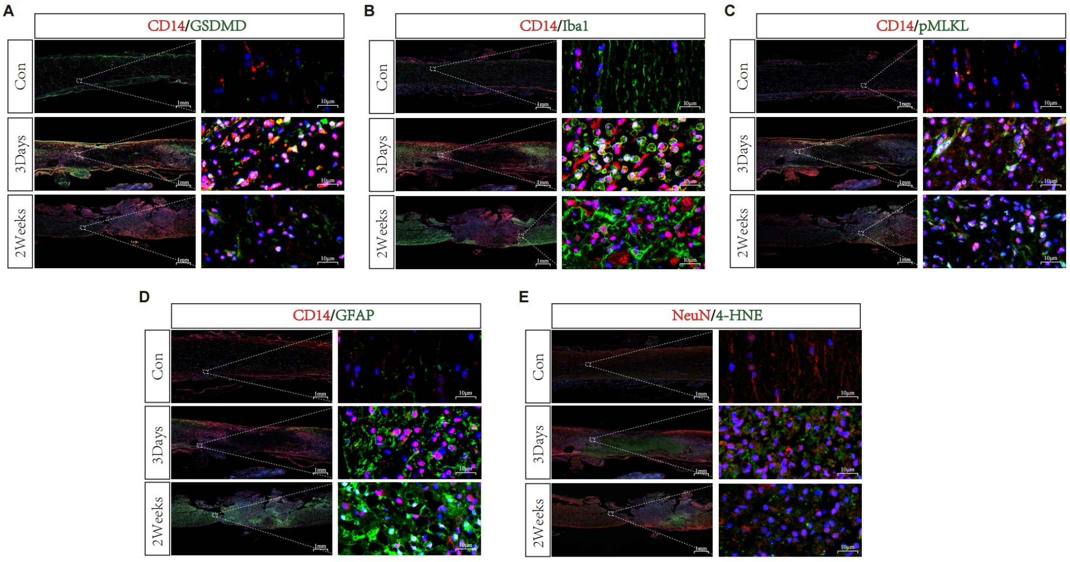

Supplementary Figure 3. Representative whole-lesion and magnified immunofluorescence images showing the spatial distribution of CD14 and lytic cell death-related markers after spinal cord injury. These images were used to show the overall lesion-level distribution and local spatial association of the indicated markers rather than to establish complete cellular morphology, cell-type-specific expression, or causal regulation.

(A) Representative immunofluorescence images showing the spatial relationship between CD14 and GSDMD in the control group and at 3 days and 2 weeks after spinal cord injury (SCI).

(B) Representative immunofluorescence images showing the spatial relationship between CD14 and Iba1.

(C) Representative immunofluorescence images showing the spatial relationship between CD14 and p-MLKL.

(D) Representative immunofluorescence images showing the spatial relationship between CD14 and GFAP.

(E) Representative immunofluorescence images showing the spatial relationship between NeuN and 4-HNE.

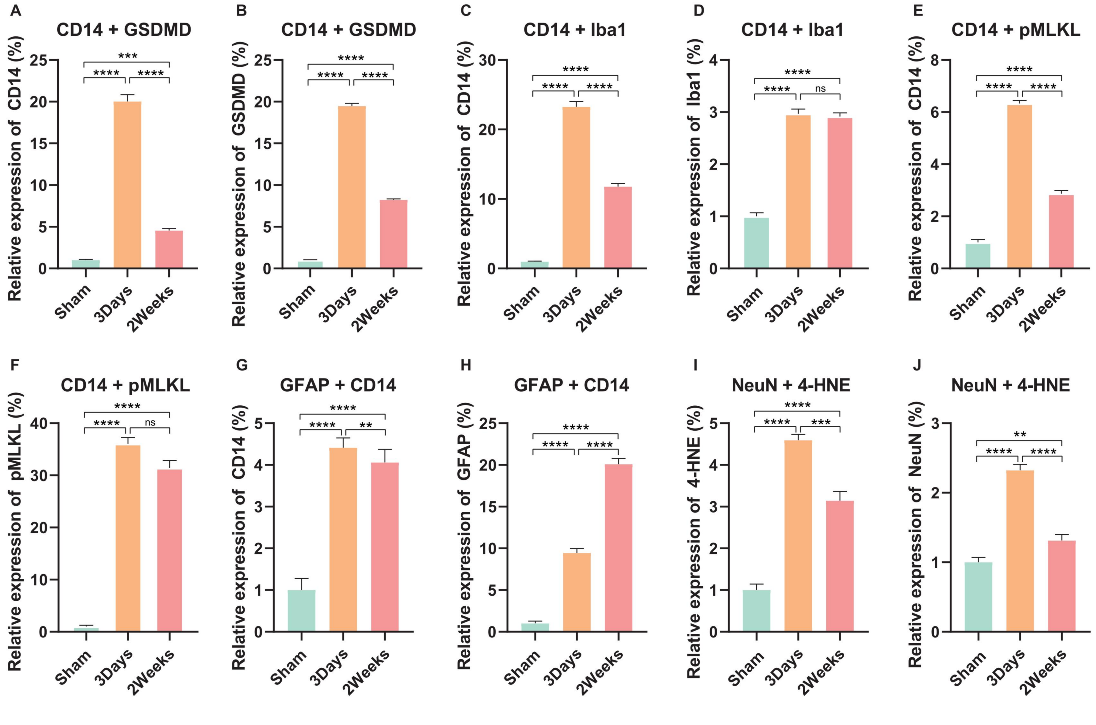

Supplementary Figure 4. Quantitative analysis of immunofluorescence signals for each marker in the Sham group and at 3 days and 2 weeks after injury.

(A, B) Relative expression levels of CD14 and GSDMD.

(C, D) Relative expression levels of CD14 and Iba1.

(E, F) Relative expression levels of CD14 and p-MLKL.

(G, H) Relative expression levels of CD14 and GFAP.

(I, J) Relative expression levels of 4-HNE and NeuN.

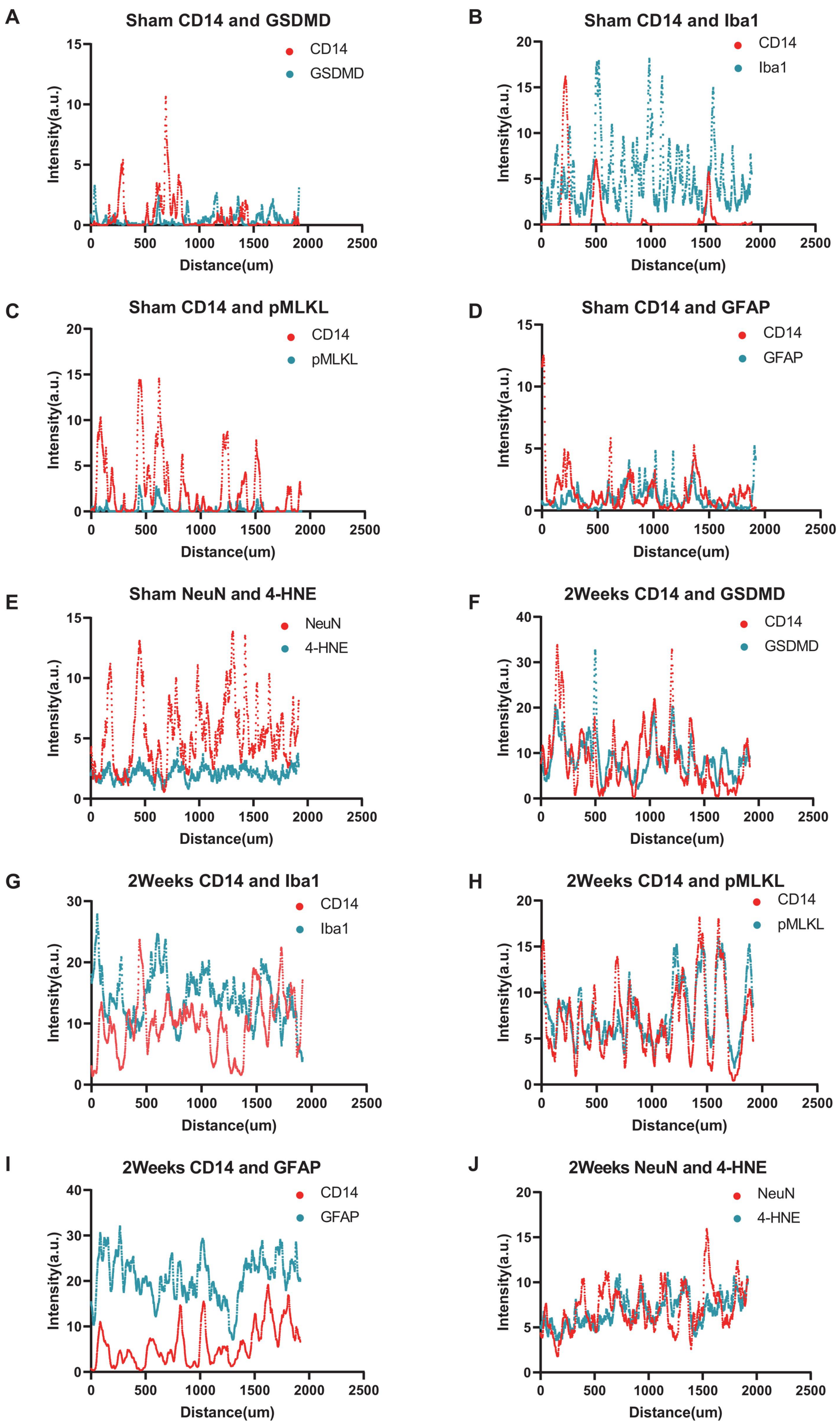

Supplementary Figure 5. Co-localization analysis in spinal cord tissues from the Sham group and at 2 weeks after injury.

(A–D) Co-localization profiles of CD14 with GSDMD, Iba1, p-MLKL, and GFAP in the Sham group.

(E) Co-localization profile of NeuN and 4-HNE in the Sham group.

(F–I) Co-localization profiles of CD14 with GSDMD, Iba1, p-MLKL, and GFAP at 2 weeks after injury.
(J) Co-localization profile of NeuN and 4-HNE at 2 weeks after injury.

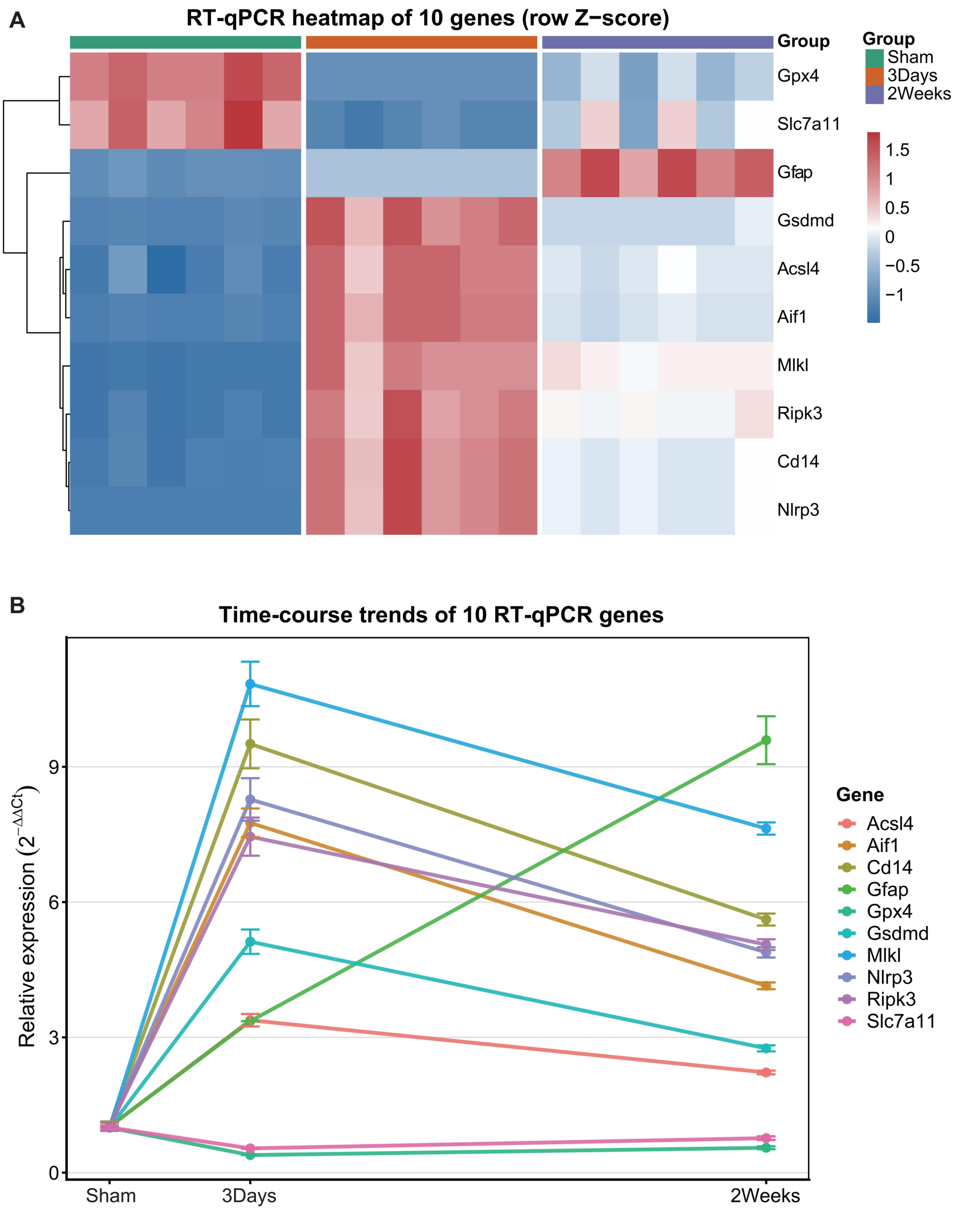

Supplementary Figure 6. Overall expression patterns of the 10 genes assessed by

RT-qPCR.

(A) Heatmap showing the expression patterns of the 10 genes in the Sham, 3-day, and 2-week groups.

(B) Temporal trend plot showing the relative expression changes of the 10 genes.

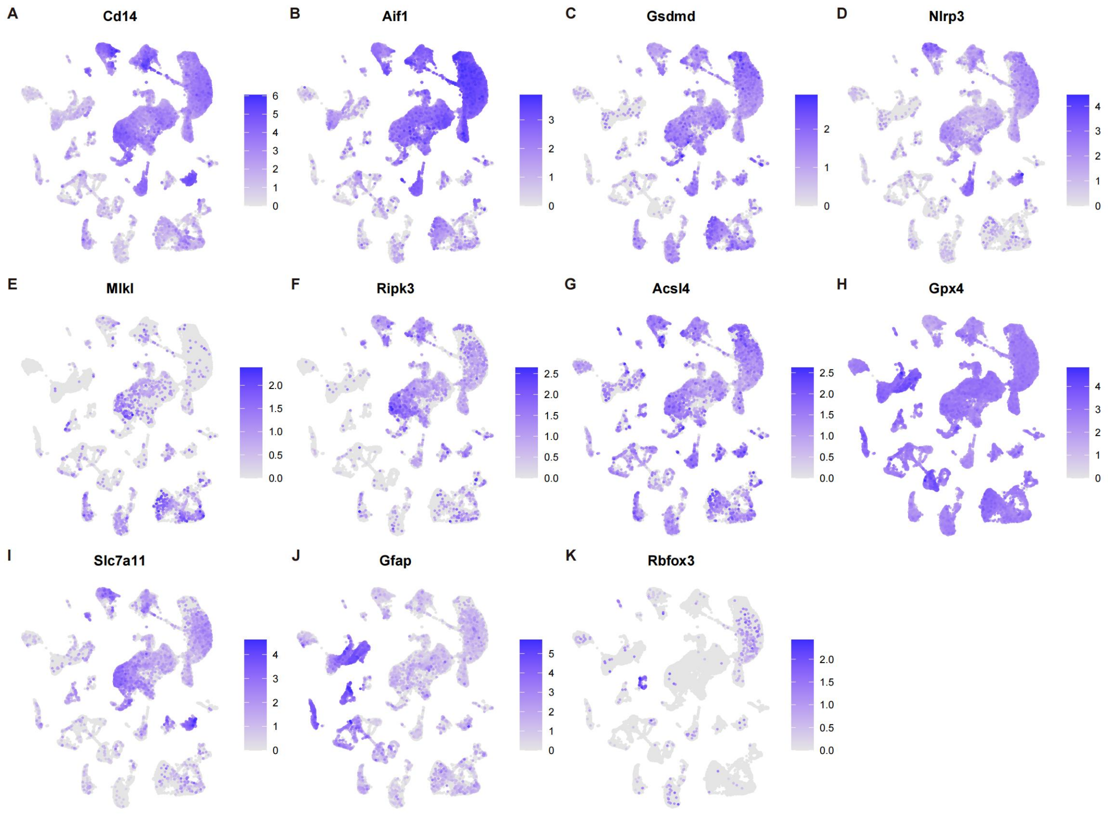

Supplementary Figure 7. Single-cell UMAP feature plots of RT-qPCR/immunofluorescence-related marker genes in GSE162610. UMAP feature plots showing the single-cell expression patterns of 11 marker genes used for RT-qPCR and immunofluorescence validation.

(A) *Cd14*, (B) *Aif1*, (C) *Gsdmd*, (D) *Nlrp3*, (E) *Mlkl*, (F) *Ripk3*, (G) *Acsl4*, (H) *Gpx4*, (I) *Slc7a11*, (J) *Gfap*, and (K) *Rbfox3*. Color intensity indicates the relative expression level of each gene in individual cells. These plots provide complementary single-cell evidence for the cellular distribution of *Cd14*, myeloid-associated genes, lytic cell death-related genes, astrocyte-associated *Gfap*, and neuron-associated *Rbfox3*.

Supplementary Table 1. Primer sequences used for RT-qPCR analysis.

Gene	Forward primer sequence	Reverse primer sequence
Cd14	TCACAATTCACTGCGGGATA	CGATGTCCTAGGAGCAAAGC
Gsdmd	CCAACATCTCAGGGCCCCAT	TGGCAAGTTTCTGCCCTGGA
Nlrp3	TCTTTGCGGCTATGTACTATCT	TTCTAATAGGACCTTCACGT
Mlkl	CCCGAGTTGTTGCAGGAGAT	TCTCCAAGATTCCATCCGCAG
Ripk3	CTGTCGCCTGCTAGAGGAAG	TCTGCTAACTTGGCGTGGAG
Acsl4	CCATATCGCTCTGTCACGCAC	CCAGGCTGTCCTTCTTCCCAAA

	TTC	C
Gpx4	ATAAGAACGGCTGCGTGGTGAAG	TAGAGATAGCACGGCAGGTCCTTC
Slc7a11	CCATCATCATCGGCACCGTCATC	TACTCCACAGGCAGACCAGAACAC
Aif1	AGCAAGGATTTGCAGGGAGG	TTGAAGGCCTCCAGTTTGGAC
Gfap	GCGAAGAAAACCGCATCACC	TCTGGTGAGCCTGTATTGGGA
